# Epidermal growth factor receptor is an essential component in E-cadherin force transduction complexes

**DOI:** 10.1242/jcs.264350

**Published:** 2025-11-13

**Authors:** Yubo Zou, Nicolas Allen, Emaan Rauf, Deborah Leckband

**Affiliations:** ^1^ Department of Biochemistry, University of Illinois at Urbana-Champaign, Urbana-Champaign, IL 61801, USA; ^2^ Departments of Chemical and Biomolecular Engineering, Bioengineering, University of Illinois, Urbana-Champaign, IL 61801, USA; ^3^ The Center for Quantitative Biology and Biophysics, University of Illinois, Urbana-Champaign, IL 61801, USA

**Keywords:** Cadherin, Epidermal growth factor receptor, Mechanotransduction, Intercellular adhesion, Integrins, Rigidity sensing

## Abstract

We present evidence that the association of the epithelial (E)-cadherin (CHD1) extracellular domain and epidermal growth factor receptor (EGFR, ErbB1) is obligatory for cadherin force transduction signaling. E-cadherin and EGFR associate at cell surfaces, independent of their cytoplasmic domains, and tension on E-cadherin activates EGFR signaling. Using engineered E-cadherin mutants that disrupt co-immunoprecipitation with EGFR, but not adhesion, we show that the hetero-receptor complex is required to mechanically activate signaling and downstream cytoskeletal remodeling at cadherin adhesions. The mutants localized the essential region on E-cadherin to domain 4 of the extracellular region (EC4). The ectodomain is also required for hetero-receptor colocalization at intercellular junctions. Although the E-cadherin mutants disrupt EGFR signaling, integrin pre-activation together with tension rescues cytoskeletal reinforcement at cadherin adhesions, confirming the role of integrins in intercellular force transduction. Furthermore, although E-cadherin suppresses EGFR-mediated proliferation, in response to extracellular matrix stiffening, the force-sensitive hetero-receptor complex regulates growth factor-dependent epithelial proliferation. These findings support the hypothesis that E-cadherin complexes with EGFR are mechano-switches at cell–cell contacts that directly couple intercellular force fluctuations to mitogen-dependent signaling.

## Teaser

**Highlighted Article:** The extracellular domain of E-cadherin, EC4, is required for EGFR association, intercellular force transduction and rigidity-dependent proliferation in dense cultures.

## INTRODUCTION

Mechanical cues orchestrate diverse biological processes, such as differentiation (Engler et al., 2006; Vining and Mooney, 2017), vascular development and homeostasis (Levesque and Nerem, 1985; Tzima et al., 2005), tumor growth (Paszek et al., 2005) and malignancy (Butcher et al., 2009). Identifying the underlying mechanisms of mechanotransduction is crucial for understanding how mechanical stimuli regulate physiological processes and for target identification. Cells receive and transduce force fluctuations at cell–matrix and cell–cell adhesions. In multicellular organisms, integrins mechanically sense the physical properties of the extracellular matrix, initiating signals that promote cell spreading (Cui et al., 2015; Pelham and Wang, 1997; Yeung et al., 2005; Zhang et al., 2008), migration (Cukierman et al., 2001; Geiger et al., 2009; Moissoglu and Schwartz, 2006; Pelham and Wang, 1997; Vogel and Sheetz, 2006) and proliferation (Dupont et al., 2011; Giancotti and Ruoslahti, 1999; Ulrich et al., 2009). Those processes in turn regulate differentiation, tissue development (Guo et al., 2006; Humphrey et al., 2014; Wozniak et al., 2003) and tumor progression (Levental et al., 2009; Paszek et al., 2005; Ulrich et al., 2009). Force transduction at cell-to-cell adhesions also regulates diverse physiological processes (Bazellières et al., 2015; Leckband and de Rooij, 2014; Pannekoek et al., 2019).

Cadherins are essential force transduction and signaling hubs at intercellular adhesions (Buckley et al., 2014; Leckband and de Rooij, 2014; Yonemura et al., 2010). These Ca^2+^-dependent, transmembrane adhesion proteins bind cadherins on adjacent cells to zip up intercellular junctions (Gumbiner, 2005; Takeichi, 1995). The intracellular region of classical cadherins is mechanically coupled to F-actin through α- and β-catenins (Buckley et al., 2014; Desai et al., 2013; Rimm et al., 1995). Through these mechanical linkages, epithelial (E)-cadherin (CHD1) complexes sense and transduce mechanical forces to regulate diverse tissue-level functions, including coordinating cell movement in epithelial tissues (Cai et al., 2014; Grimaldi et al., 2020; Ladoux et al., 2015; Tambe et al., 2011) and stabilizing germ-layer organization (Krieg et al., 2008; Maître et al., 2012; Slováková et al., 2022). E-cadherin is also under constitutive tension and is thus capable of sensing polarized forces to align cell division during morphogenesis (Borghi et al., 2012; Hart et al., 2017). Because cadherins are essential to these processes, identifying molecular mechanisms of cadherin force transduction is key to understanding how mechanical cues regulate physiology.

Different E-cadherin force transduction mechanisms have been identified. One involves the actin-binding protein, α-catenin (herein referring to CTNNA1), which mechanically couples E-cadherin and actin. Tension on E-cadherin complexes unfurls α-catenin, to expose a vinculin-binding site, which recruits vinculin and actin and mechanically reinforces the junctions (Kim et al., 2015; Le Duc et al., 2010; Yonemura et al., 2010). α-Catenin unfurling does not activate signaling, but ligand-specific E-cadherin tugging activates EGFR and kinases such as AMP-activated protein kinase (AMPK), phosphatidylinositol 3-kinase (PI3K) and Abl kinase (Bays et al., 2017; Muhamed et al., 2016; Sehgal et al., 2018; Sullivan et al., 2022). The co-immunoprecipitation of EGFR and E-cadherin is disrupted by cadherin tugging forces, to initiate EGF-dependent signaling (Sehgal et al., 2018; Sullivan et al., 2022). Signals downstream from EGFR activate integrins, which in turn increase cell contractility and regulate the recruitment of vinculin and actin to stressed cadherin adhesions (Kong et al., 2022; Muhamed et al., 2016; Tzima et al., 2005). Tension on E-cadherin might also regulate ATP production via AMPK through LKB1 (also known as STK11) (Bays et al., 2017). Although several studies have documented force-activated α-catenin unfurling and vinculin recruitment (Kim et al., 2015; Le Duc et al., 2010; Seddiki et al., 2018; Yonemura et al., 2010), much less is known about signal initiation and the ensuing cascades.

There are several examples of crosstalk between E-cadherin and EGFR (Ramírez Moreno and Bulgakova, 2022; Schwartz and DeSimone, 2008; Weber et al., 2011). For example, EGFR activation destabilizes cadherin adhesions and facilitates multicellular rearrangements during wound healing and tissue remodeling (Chiasson-MacKenzie and McClatchey, 2018). E-cadherin adhesion suppresses EGFR signaling (Bazellières et al., 2015; Mendonsa et al., 2018; Perrais et al., 2007; Qian et al., 2004) during contact inhibition of proliferation (CIP). Matrix stiffening and E-cadherin junction disruption increase EGF-dependent proliferation in dense cultures (Kim and Asthagiri, 2011; Maruthamuthu et al., 2011).The coordination between mechanics, growth factor receptor activity and E-cadherin influences cell differentiation and polarization across stratified epidermis (Rübsam et al., 2017). EGFR activation following E-cadherin adhesion increases cortical tension to accelerate the expansion of intercellular contacts (Fu et al., 2022).

Identifying mechanisms by which cadherin mechanically regulates EGFR is therefore central for understanding how these receptors regulate physiology and for identifying targets that could disrupt or enhance the interactions. The receptors might interact indirectly, by controlling cell mechanics or signaling. Other evidence suggests that E-cadherin and EGFR associate through their extracellular regions, and missense mutations in the E-cadherin extracellular region correlated with increased EGFR activity (Fedor-Chaiken et al., 2003; Mateus et al., 2007; Qian et al., 2004). Fluorescence measurements have shown that tailless E-cadherin and EGFR associate on the plasma membranes of live cells (Sullivan et al, 2022). However, there is also evidence that E-cadherin associates with EGFR through cytosolic binding partners. The protein neurofibromatosis type 2 (NF2) tumor suppressor protein Merlin binds α-catenin (Curto et al., 2007; Gladden et al., 2010), and the Na^+^/H^+^ exchanger regulatory factor (NHERF) couples Merlin to EGFR (Curto et al., 2007). Merlin might interact with actin, raising the possibility that the latter complex could also mechanically regulate signaling (Bazellières et al., 2015).

This study tested the hypothesis that the assembly of E-cadherin–EGFR complexes is required to mechanically activate EGFR signaling at intercellular adhesions. Here, E-cadherin mutants were engineered to disrupt EGFR interactions, but not adhesion. Mechanical and biochemical measurements explored how disrupting the complex impacts force transduction and identified a key E-cadherin region required for hetero-receptor complex assembly and mechanotransduction. We further tested the hypothesis that the hetero-receptor complexes mechanically regulate EGF-dependent epithelial proliferation, in response to matrix stiffening.

## RESULTS

### E-cadherin extracellular domain 4 is required for EGFR complex formation

**Fig. 1.**
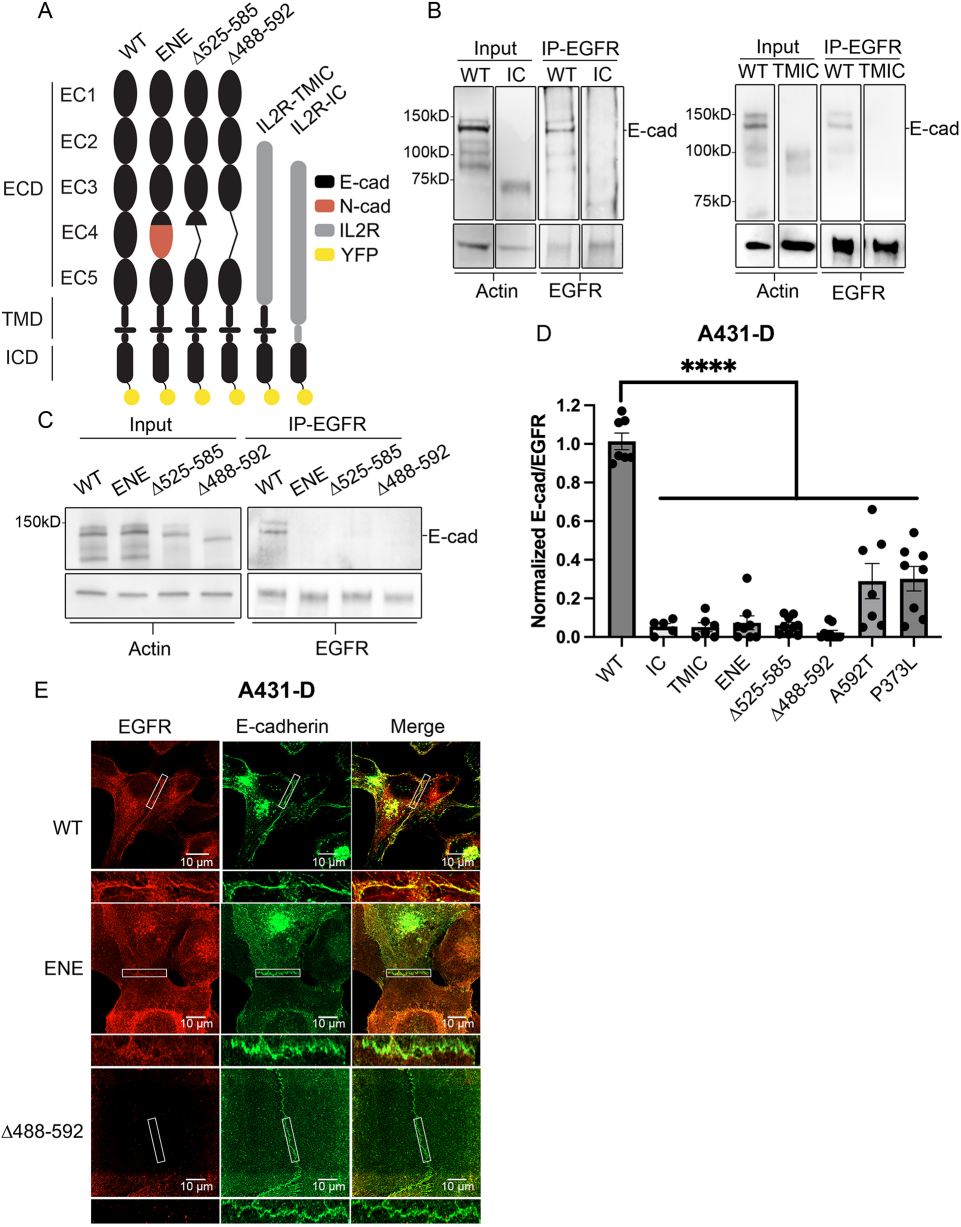
**Extracellular domain 4 of E-cadherin is required for epidermal growth factor receptor binding.** (A) Schematic of engineered E-cadherin mutants. TMD and ICD are the transmembrane and intracellular domains, respectively. (B) Western blot of cadherin (input) and EGFR co-IP results obtained with A431-D cells expressing WT E-cadherin or the IC (left) or TMIC (right) mutants shown in A. EGFR immunoprecipitants (IP-EGFR) were probed for EGFR and E-cadherin. Input, 2%. (C) EGFR co-IP results obtained with A431-D cells expressing WT, ENE, Δ525-585 or Δ488-592 mutants. Input, 2%. (D) Normalized ratios for E-cadherin/EGFR levels obtained from co-IP results from B and C. Results are also shown for point mutants P373L and A592T. Ratios are normalized to the WT E-cadherin expressing cells. Data are reported as the mean±s.e.m. (*N*≥5 independent experiments). ****P<0.0001 (two-tailed unpaired Student's *t*-test). (E) Representative (of three experimental repeats and 30 imaged junctions) super resolution fluorescence images of WT, ENE, or Δ488-592 E-cadherin variants and EGFR at junctions between A431-D cells. The white boxes indicate regions of interest at E-cadherin junctions. Scale bars: 10 μm.

The selection of E-cadherin mutations used in this study was guided by prior findings that implicated the extracellular domain, the transmembrane domain or the intracellular domain in different cadherin–receptor-tyrosine-kinase (RTK) interactions (Coon et al., 2015; Curto et al., 2007; Fedor-Chaiken et al., 2003; Qian et al., 2004; Sullivan et al., 2022; Williams et al., 2001). The engineered mutants are in Fig. 1A. To test the role of the extracellular (EC) and transmembrane (TM) domains, E-cadherin segments were exchanged with the corresponding regions of the interleukin 2 receptor (IL2R, specifically ILR2A). We designate the chimera with the extracellular domain or extracellular-transmembrane region as, respectively, IL2R-TMIC and IL2R-IC. The EC4 deletion mutants Δ525–585 and Δ488–592 included partial and full domain deletions, respectively. In the ENE chimera, EC4 of E-cadherin was exchanged with the same region of N-cadherin (CDH2). The latter chimera alters the epithelial-to-mesenchymal transition (Kim et al., 2000). In addition, the two engineered point mutants P373L and A592T have been previously reported to alter EGFR interactions with E-cadherin (Mateus et al., 2009; Petrova et al., 2016). The mutants were expressed in E-cadherin-null A431-D and E-cadherin-knockout MCF 10A cells (MCF 10A KO; gift from Barry Gumbiner, University of Virginia, Charlottesville, USA). The A431-D line is an E-cadherin-null subclone of the EGFR-overexpressing A431 line (Lewis et al., 1997). The stably transfected cells were sorted for surface expression levels that were similar to those in A431 cells (30–36 cadherin molecules/µm^2^; Table S1) or to those in parental MCF 10A cells (∼30 cadherin molecules/µm^2^; Table S1).

To ensure that force transduction differences were due to disrupted E-cadherin–EGFR interactions, and not cadherin affinity differences, studies only used mutants that did not significantly alter E-cadherin adhesion. The exception is the IL2R chimera, which lacks the cadherin adhesive domain EC1. Capillary flow assays quantified the relative adhesion of wild type (WT) and E-cadherin mutants, based on the fluid shear stress needed to detach 50% of the cells from capillary walls (WSS_50_) coated with either soluble E-cadherin-Fc extracellular domains or with anti-IL2R antibody.

Control A431-D cells, which do not express E-cadherin, detached at low shear stress (Fig. S1A). Similarly, IL2R-IC expressing cells did not adhere to E-cadherin-coated surfaces. However, cells expressing WT E-cadherin, ENE or Δ525–585 adhered similarly to E-cadherin-coated capillaries (Fig. S1A). Quantitative comparisons are in Fig. S1B. The point mutant A592T and the full EC4 domain deletion mutant Δ488–592 also adhered similarly to WT E-cadherin. Cells expressing the P373L mutant exhibited a slightly lower WSS_50_ value than WT E-cadherin (Fig. S1C,D). Because the cadherin expression levels were similar, the relative adhesion reflects the cadherin bond strengths. The higher IL2R-IC adhesion to antibody-coated capillaries can be attributed to strong antibody–IL2R binding. The latter also confirms that the antibody binds robustly to the IL2R domain of the E-cadherin chimeras.

### E-cadherin EC4 mutations disrupt EGFR co-immunoprecipitation

E-cadherin co-immunoprecipitation (co-IP) measurements tested how the E-cadherin mutations (Fig. 1A) alter EGFR interactions. WT E-cadherin co-immunoprecipitated with EGFR but exchanging the extracellular domain or the extracellular and transmembrane domains with the corresponding IL2R regions abolished co-IP with EGFR. The IL2R-IC chimera expression was slightly lower than WT E-cadherin or IL2R-TMIC but results with both constructs showed that replacing the extracellular domain alone was sufficient to abolish the E-cadherin–EGFR interaction (Fig. 1B).

Studies with domain mutants further localized the possible binding interface. Results obtained with the EC4 domain swap (ENE) and with the EC4 domain deletion mutants Δ525–585 and Δ488–592 are in Fig. 1C. Similar expression levels (Table S1) enabled semi-quantitative comparisons of co-IP levels. Deleting or exchanging EC4 abolished the EGFR association, thus demonstrating that EC4 is crucial for hetero-receptor complex formation. The point mutations P373L and A592T, which flank the EC4 domain, attenuated but did not abolish EGFR co-IP (Fig. 1D). Fig. 1D compares the quantitative results, when normalized by co-IP levels for WT E-cadherin. These results show that the extracellular region of E-cadherin mediates hetero-receptor complex formation, and the EC4 domain is crucial for this interaction. The cytoplasmic region does not detectably impact complex assembly, as all mutants possess intact cytodomains.

### EGFR does not colocalize with E-cadherin mutants at intercellular junctions

In cells expressing WT E-cadherin, EGFR colocalizes with cadherin at intercellular junctions. This association results in reduced EGFR mobility (Curto et al., 2007), and E-cadherin adhesion correlates with a lower, population-average EGF affinity for EGFR (Qian et al., 2004). Super resolution Airyscan immunofluorescence imaging confirmed that E-cadherin and EGFR colocalize at junctions (Fig. 1E; Fig. S1E), based on the Pearson's correlation coefficient. Remarkably, EGFR was almost completely excluded from junctions when either ENE or Δ488–592 mutants were expressed, despite EGFR overexpression in A431-D cells (Fig. 1E). The overlap of E-cadherin and EGFR immunofluorescence at junctions (Fig. S1F) confirmed that WT E-cadherin and EGFR colocalize. By contrast, the distribution of E-cadherin mutants ENE or Δ488–592 did not overlap with EGFR (Fig. 1E), even though both proteins are capable of binding cytoplasmic linkers. This behavior is not cell type dependent, as results with MCF 10A KO cells rescued with either WT E-cadherin or Δ488–592 were similar (Fig. S1E,F).

### Hetero-receptor complexes are required for force transduction

**Fig. 2.**
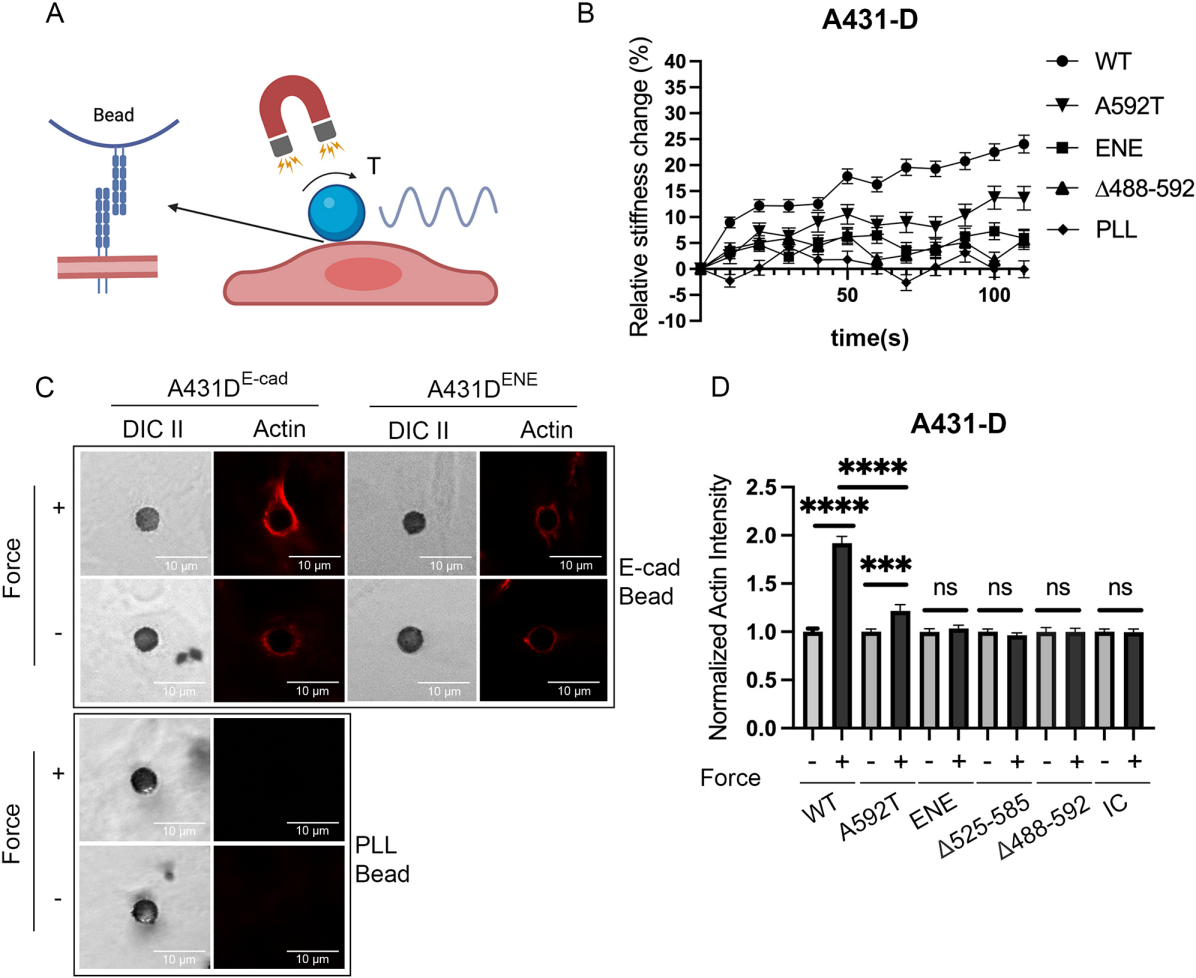
**E-cadherin force transduction and actin recruitment requires E-cadherin/EGFR association.** (A) Schematic of the MTC set up. Ferromagnetic beads modified with E-cadherin-Fc fragments bind to cadherins on the cell surface (left). An oscillating magnetic field generates a twisting torque, T, on the bead and bead–cell interface (right). (B) Relative change in cell stiffness (%) versus the bead twisting time(s) measured with A431-D cells expressing WT E-cadherin or mutants A592T, ENE or Δ488-592. Control beads were coated with PLL. Data shown are the mean±s.e.m. determined from analyses of *n*>150 beads from *N*=3 independent experiments. (C) Representative DIC and immunofluorescence images of actin at beads without (top) or following (bottom) applying force on beads. Data shown were obtained with A431-D cells expressing WT E-cadherin (A431D^E-cad^) or ENE (A431D^ENE^). Controls with PLL beads are also shown. (D) Normalized mean fluorescence intensity of actin within regions of interest 1 μm from the bead edge with (+) or without (−) force. Data were obtained with A431-D cells expressing WT E-cadherin or A592T, ENE, Δ525-585 or IC mutants. All data are normalized to the no-force condition and are shown as the mean±s.e.m. (*n*>100 beads, *N*=3 independent measurements). ****P<0.0001; ***P<0.001; *P<0.05; ns, not significant (two-tailed unpaired Student's *t*-test).

Magnetic twisting cytometry (MTC) measurements tested the hypothesis that E-cadherin force transduction requires hetero-receptor complex formation (Fig. 2A). In these measurements, ferromagnetic beads coated with E-cadherin-Fc apply an oscillating (shear) force on *trans* bonds between cadherins on the cell surface and E-cadherin on the beads (Wang et al., 1993). MTC quantifies force-activated changes in the elastic modulus of the bead–cell junctions. Cadherin force transduction triggers an increase in the measured stiffness (‘adaptive stiffening’), which is due to: (1) myosin-dependent cell contractility and (2) vinculin (and actin) recruitment to stressed cadherin receptors (Barry et al., 2014; Le Duc et al., 2010).

Fig. 2B and Fig. S2A show cell stiffening, as a function of bead twisting time, measured with both A431-D cells and MCF 10A KO cells that stably express WT E-cadherin or mutants. Tugging on WT E-cadherin triggered an increase in bead–junction stiffness (Fig. 2B), but negative controls with poly-L-lysine (PLL)-conjugated beads did not. Tugging on the IL2R-IC and IL2R-TMIC mutants with anti-IL2R antibody-coated beads failed to activate stiffening, within the limits of error. The quantitative relative changes in cell stiffness are in Table S2.

Studies then tested the impact of extracellular domain mutations on force transduction. Despite exhibiting similar adhesion strengths (Fig. S1A–D), stiffening measured with A431-D cells expressing ENE, Δ525-585 or Δ488-592 mutants were all significantly lower than in cells expressing WT E-cadherin, but were similar to each other (Fig. 2B, Fig. S2B). The stiffening response of MCF 10A KO cells expressing Δ488–592 was also significantly lower than WT E-cadherin-expressing cells (Fig. S2A,C). The point mutants attenuated, but did not abolish, adaptive stiffening (Fig. 2B; Fig. S2B, Table S2). The stiffness change measured with P373L-expressing A431-D cells was similar to that found with A592T, and the stiffening responses of both mutants were statistically different from WT E-cadherin (Fig. S2B,C; Table S2). Together with the co-IP data, these MTC results support the hypothesis that force transduction requires hetero-receptor complex formation.

In MTC measurements, cadherin force transduction activates vinculin and actin recruitment to E-cadherin beads (Barry et al., 2014; Le Duc et al., 2010). Confocal immunofluorescence imaging was hence used to quantify actin accumulation at stressed bead–cell junctions to test whether EGFR uncoupling disrupts force-dependent actin accumulation. Fig. 2C shows the quantified changes in the mean fluorescence intensity (MFI) of actin around beads on A431-D cells expressing WT E-cadherin or the ENE mutant, with and without applied force. With WT E-cadherin, bead twisting stimulated actin accumulation relative to no-force controls. Control PLL beads did not recruit actin (Fig. 2C). By contrast, none of the mutants ENE, Δ525–585, Δ488–592 or IL2R-IC triggered detectable changes in actin relative to no-load controls (Fig. 2D). However, tugging on the A592T mutant did activate actin recruitment, but the change was lower than at WT E-cadherin adhesions (Fig. 2D).

### EGFR activation by E-cadherin tugging

To test the hypothesis the mechanical activation of EGFR requires both association with E-cadherin and EGF, Western blots were used to quantify the levels of phosphorylation of EGFR Y845 (pY845) after E-cadherin tugging. Cadherin tugging increases phosphorylation at tyrosine 845 (Y845), in the presence of EGF (Sato et al., 1995; Sullivan et al., 2022). In serum-starved A431-D cells expressing WT E-cadherin without added EGF, pY845/EGFR levels were low, and bead twisting had no significant effect on EGFR phosphorylation (Fig. S3A). Fig. S3B shows the quantified pY845/EGFR ratios, normalized to that in unperturbed serum-starved cells. Including 3 nM EGF increased the normalized pY845/EGFR ratio, and tugging on cadherin further increased the pY845/EGFR level relative to the no-load condition with EGF (Fig. S3B). This behavior agrees with prior findings (Sullivan et al., 2022).

**Fig. 3.**
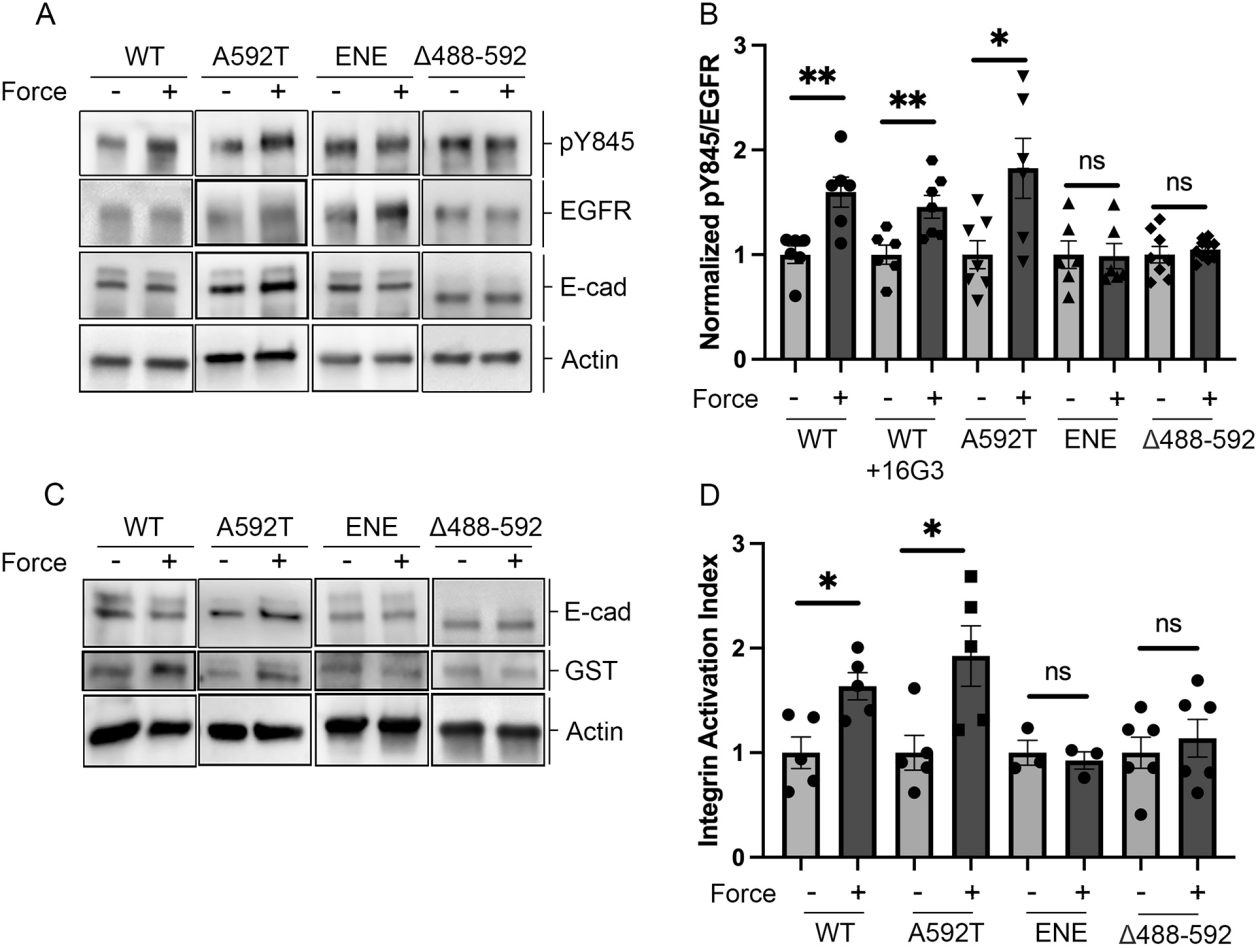
**E-cadherin mutants disrupt the mechanical activation of EGFR and downstream integrins.** (A) Representative western blots of pY845, EGFR, E-cadherin, and actin measured with A431-D cells expressing WT E-cadherin or mutants A592T, ENE and Δ488–592 with (+) or without (−) force on cadherin bonds. (B) Normalized pY845/EGFR ratios determined from western blots as in A. Data are normalized to the no-force condition in each case. 16G3 antibody blocks the formation of new integrin adhesions. Data are mean±s.e.m. (*N*≥3 independent experiments). (C) Western blots of E-cadherin, GST–FNIII_9-11_ (GST) and actin in A431-D cells expressing WT E-cadherin or mutants A592T, ENE and Δ488-592. Measurements were done with or without force on cadherin bonds. (D) Quantified GST intensity (integrin activation index), with or without applied force. Data are the mean±s.e.m. (*N*≥3 independent experiments( Results are normalized to the no-force condition in each case. **P<0.005; *P<0.05; ns, not significant (two-tailed unpaired Student's *t*-test).

We next tested whether the E-cadherin mutants could mechanically activate EGFR. Measurements were done with cells in 10% serum, which is commonly used for MTC measurements (Barry et al., 2014; Le Duc et al., 2010). The average EGF concentration in 10% serum is ∼1 nM, and the lower basal activity in serum would be expected to increase the dynamic range of the response. Under these conditions (no additional EGF), tugging on WT E-cadherin increased pY845/EGFR levels relative to that in no-load controls (Fig. 3A,B). Western blots of pY845 and EGFR in A431-D cells expressing the ENE or Δ488–592 mutants are in Fig. 3A, and the quantified pY845/EGFR ratios, normalized to the no-force condition in each case, are in Fig. 3B. Tugging on either the ENE or Δ488–592 mutant had no effect on EGFR phosphorylation. However, tugging on the A592T mutant increased the normalized pY845/EGFR levels relative to the no-force condition (Fig. 3A,B). The A592T mutant response is similar to that of WT E-cadherin and indicates that, despite weakening the hetero-receptor complex, the residual interaction is sufficient to transduce force and activate EGFR. The results support the hypothesis that E-cadherin–EGFR association is required to mechanically initiate EGFR signaling.

Integrins also activate EGFR, independently of EGF (Saxena et al., 2017), and integrins are stimulated downstream of cadherin force transduction (Muhamed et al., 2016; Sehgal et al., 2018). To verify that EGFR phosphorylation following E-cadherin tugging was not caused by integrins, cells were treated with the fibronectin-blocking antibody 16G3, which prevents the formation of new integrin adhesions. Although 16G3 disrupts cell stiffening and actin recruitment (Sullivan et al., 2022), it had no impact on increased pY845/EGFR levels stimulated by WT E-cadherin relative to no-force controls (Fig. 3B). Thus, the increased EGFR phosphorylation in these assays is due to E-cadherin and not to integrins.

To test whether hetero-receptor complex formation is required to mechanically initiate cascades that activate downstream integrins, we used a GST-tagged fragment of fibronectin domains 9–11 (GST–Fn_9-11_), which binds activated α5β1 integrin (Bouaouina et al., 2008; Coon et al., 2015; Ramos and DeSimone, 1996). Fig. S3C shows a Western blot of GST–Fn_9-11_ incorporation in A431-D cells expressing WT E-cadherin, with or without applied force. The quantitative results are in Fig. S3D. E-cadherin tugging increased GST–Fn_9-11_ incorporation, relative to no-force controls, but 16G3 treatment abolished the probe uptake (Fig. S3C,D). Prior studies have also suggested that adaptive stiffening requires integrin activation, and both 16G3 and an anti-α5 blocking antibody abolished stiffening in A431-D cells expressing WT E-cadherin (Fig. S3E).

Western blots of force-activated GST–Fn_9-11_ uptake in cells expressing ENE, Δ488–592 or A592T mutants are in Fig. 3C, and quantitative results are in Fig. 3D. In contrast to WT E-cadherin, tugging on the ENE or Δ488-592 mutants did not activate integrins. The point mutant A592T did trigger significant integrin activation, consistent with increased pY845 levels and cell stiffening. These results support the hypothesis that E-cadherin–EGFR complexes are required to mechanically initiate signals that activate downstream integrins and cell stiffening.

### Integrin activation rescues cell stiffening and actin recruitment in cells expressing E-cadherin mutants

**Fig. 4.**
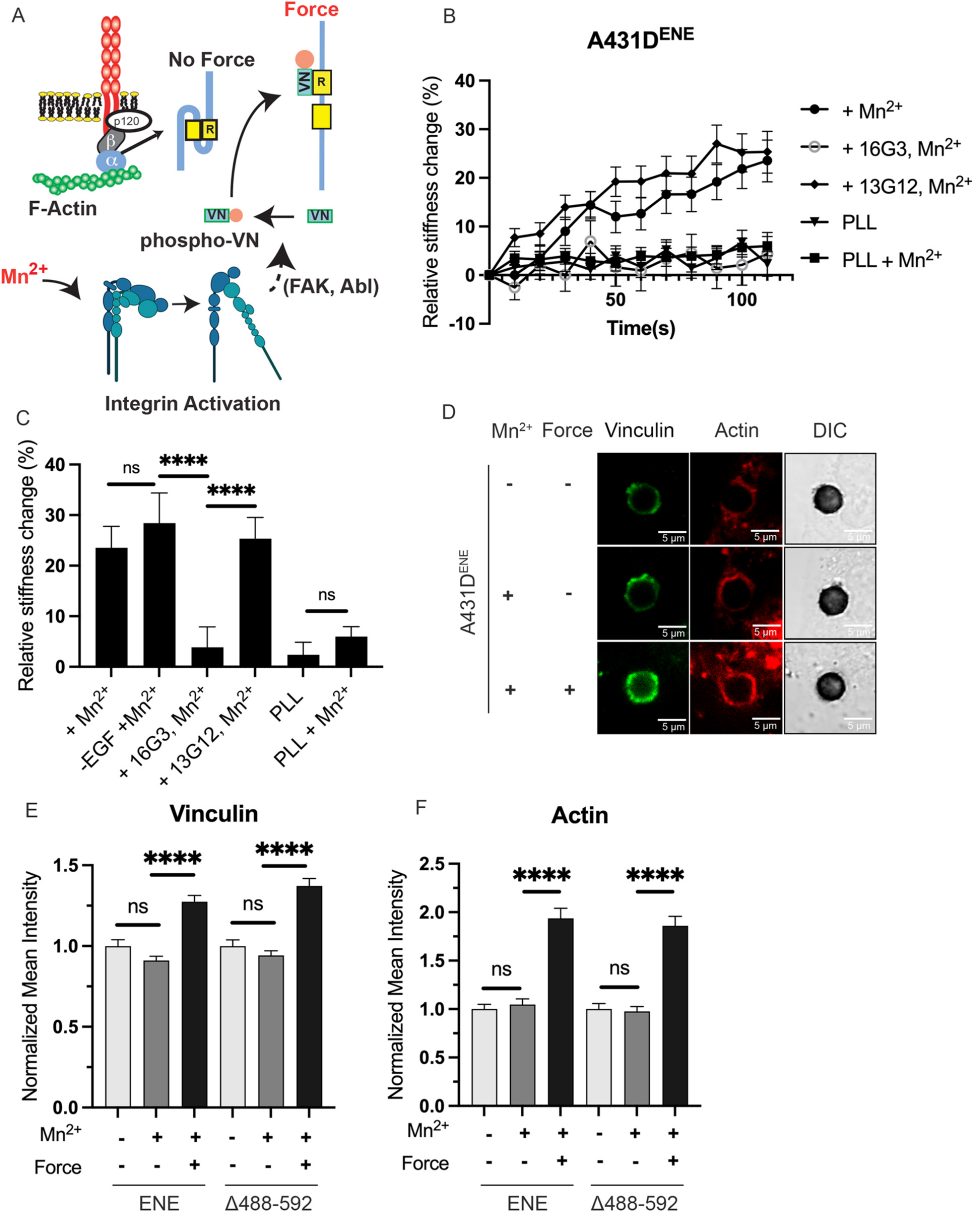
**Integrin pre-activation rescues E-cadherin force transduction signatures when EGFR–E-cadherin complexes are disrupted.** (A) Schematic of the experimental setup. Mn^2+^ treatment activates integrins, which activate FAK and downstream Abl kinase, which phosphorylates vinculin (VN). Phosphorylated vinculin is recruited to α-catenin. Force on cadherins is required to unfurl α-catenin, so it can bind vinculin. (B) Relative stiffness changes (%) versus twisting time measured with A431-D cells expressing ENE. Cells were pre-treated with Mn^2+^ prior to bead twisting. Mn^2+^-treated cells were also treated with the fibronectin blocking antibody 16G3 and with the isotype control 13G12. Controls were done with PLL coated beads with or without Mn^2+^. Data are the mean±s.e.m. (number of beads, *n*>150, *N*=3 independent experiments). (C) Final percentage (%) stiffness change, after 2 min of bead twisting for conditions shown in B. (D) Representative DIC and confocal immunofluorescence images of vinculin and actin in regions of interest around beads. Measurements were done with A431-D cells expressing the ENE mutant, with or without applied force and with or without Mn^2+^ pre-treatment. (E,F) Normalized fluorescence intensity of (E) vinculin and (F) actin in regions of interest within 1 µm of the bead edge. All data were normalized to the no-Mn^2+^ and no-force condition and are the mean±s.e.m. (number of beads, *n*>100, *N*=4 independent experiments). ****P<0.0001 ns, not significant (two-tailed unpaired Student's *t*-test).

In cadherin force transduction, integrins are activated downstream from EGFR (Muhamed et al., 2016; Tzima et al., 2005), but prior studies have also shown that integrin activation also facilitates vinculin and actin recruitment to stressed cadherin complexes. Integrins activate focal adhesion kinase (FAK; also known as PTK2) and RhoA (Kong et al., 2022; Sehgal et al., 2018). FAK stimulates Abl kinase, which directs vinculin to cadherin junctions (Bays et al., 2014), and RhoA has been found to regulate cell contraction and actin accumulation at stressed adhesions (Collins et al., 2012; Sehgal et al., 2018). The latter findings suggested that integrins are key EGFR effectors that couple cadherin-initiated signals to cytoskeletal remodeling at junctions. We hypothesized that the lack of adaptive stiffening and actin accumulation at E-cadherin beads (see Fig. 2B,C) bound to cells expressing some mutants were due to the inability of the E-cadherin mutants to initiate EGFR signals needed to activate integrins. Based on this model, the activation of integrins by a different mechanism (see Fig. 4A, bottom), such as Mn^2+^ treatment, in conjunction with E-cadherin tugging, might restore vinculin recruitment and actin remodeling. Gain of function studies thus tested whether integrin pre-activation with Mn^+2^ could rescue adaptive stiffening and cytoskeletal remodeling, in mutant expressing cells. Importantly, in the absence of force, α-catenin is autoinhibited and cannot bind vinculin (Bazellières et al., 2015; Kim et al., 2015; Le Duc et al., 2010; Yonemura et al., 2010), so measurements were done with and without E-cadherin tugging, which is required to unfurl α-catenin (Fig. 4A, top).

Tugging on the ENE mutant with E-cadherin beads did not trigger cell stiffening (see Fig. 2B), but Mn^2+^ pre-treatment together with E-cadherin tugging resulted in a force-dependent increase in cell stiffness (Fig. 4B). The response is similar to that in untreated cells that express WT E-cadherin and statistically higher than untreated ENE-expressing cells. PLL-coated beads (controls) did not activate cell stiffening, with or without Mn^2+^ pre-treatment (Fig. 4B,C). The fibronectin blocking 16G3 antibody abolished stiffening by Mn^2+^-treated ENE-expressing cells, but the isotype control 13G12 had no effect (Fig. 4B,C). These results support the hypothesis that integrins are key downstream EGFR effectors in the cadherin force transduction cascade. They also confirm that uncoupling E-cadherin–EGFR complexes breaks the signaling link between cadherin tension and downstream integrin activation, which facilitates cadherin junction remodeling.

E-cadherin force transduction requires EGF, and serum starvation ablates cell stiffening (Sehgal et al., 2018; Sullivan et al., 2022). In serum-starved cells that expressed the ENE mutant, pre-activating integrins similarly restored cell stiffening (Fig. 4C). Data obtained with the Δ488–525 mutant were qualitatively similar (Fig. S4A). Thus, integrin pre-activation together with applied cadherin tension rescues cadherin-mediated adaptive stiffening, in the absence of EGF.

Measurements with the GST–Fn_9-11_ reporter confirmed that the changes following Mn^2+^ treatment were due to integrin activation. Fig. S4B shows western blots of GST–Fn_9-11_ in cells expressing the ENE mutant, and Fig. S4C summarizes the quantified results obtained with cells expressing ENE or Δ488–592 mutants. The increased probe labeling following Mn^2+^ treatment, relative to untreated controls, confirms integrin activation, but 16G3 treatment blocked GST–Fn_9-11_ incorporation in ENE-expressing cells. These controls confirmed that Mn^2+^ activates integrins.

We next tested whether integrin pre-activation by Mn^2+^ treatment also rescues actin remodeling at junctions in cells expressing cadherin mutants. To ensure that α-catenin unfurls to expose the vinculin-binding site, measurements combined tugging on E-cadherin with Mn^2+^ treatment (Fig. 4A). In the absence of tension on cadherin and α-catenin, Mn^2+^ had no detectable effect on vinculin or actin levels in cells expressing either the ENE or Δ488–592 variants. However, the combination of force and Mn^2+^ rescued vinculin (Fig. 4D,E) and actin (Fig. 4D,F) recruitment to mutant ENE adhesions. The immunofluorescence results were validated in pull-down measurements with E-cadherin-coated beads. Western blots of proteins pulled down with beads (Fig. S4D) show that both Mn^2+^ and force are required to rescue actin (Fig. S4E) and vinculin (Fig. S4F) recruitment. Western blots of normalized pY845/EGFR levels confirmed that the restored stiffening and cytoskeletal remodeling were due to integrin activation, rather than to EGFR signaling: there was no significant change in the pY845/EGFR ratios after Mn^2+^ treatment of A431-D cells expressing either ENE or Δ488–592 mutants (Fig. S4G,H). Thus, with E-cadherin mutants that disrupt the signaling link between tension and downstream integrin effectors, integrin pre-activation restores actin remodeling at cadherin adhesions.

### The E-cadherin extracellular domain regulates EGFR signaling in confluent monolayers

E-cadherin adhesion reduces the apparent affinity of EGF for EGFR and suppresses EGFR signaling in confluent monolayers (Lichtner and Schirrmacher, 1990; Mendonsa et al., 2018; Qian et al., 2004; Takahashi and Suzuki, 1996), but increased tension on cadherin adhesions activates EGFR and MAPK signaling (Sullivan et al., 2022). We next tested how the E-cadherin mutants alter EGFR signaling in confluent monolayers by quantifying differences in EGF-dependent EGFR activation in cells expressing mutants versus in those expressing WT E-cadherin. We compared western blots of phosphorylated EGFR (pY845) in monolayers on fibronectin-coated glass (Figs. S5A,B). Relative to E-cadherin-null cells exposed to the same EGF concentrations, WT E-cadherin expression reduced pY845/EGFR ratios in both MCF 10A KO (Fig. S5A) and in A431-D (Fig. S5B) cells. At higher EGF concentrations, we observed lower total EGFR levels (Fig. S5A,B), which we attribute to receptor internalization (Savio et al., 2016). In sub-confluent monolayers, the expression of WT E-cadherin had no detectable influence on EGFR phosphorylation relative to that seen in E-cadherin-null cells (Fig. S5C).

We then tested the capacity of E-cadherin mutants to suppress EGFR signaling in dense cultures by comparing pY845/EGFR ratios in serum-starved A431-D monolayers expressing different cadherin mutants, with or without added 3 nM EGF (Fig. S5D). The EGF addition increased pY845 levels in all cells (Fig. S5D,E). In parental A431-D cells, pY845/EGFR ratios were higher than in cells expressing WT E-cadherin (Fig. S5E). Compared to cells expressing WT E-cadherin, pY845/EGFR values were also higher in cells expressing mutants ENE, Δ525–585, Δ488–592, IC, TMIC, P373L and A592T (Fig. S5E). The difference between the mutants and WT E-cadherin is significant, but the statistical power was not sufficient to distinguish among the mutants.

E-cadherin-Fc-coated beads were also used to determine how E-cadherin mutant ligation affects EGFR activation, which is suppressed by homophilic WT E-cadherin adhesion (Perrais et al., 2007). Cells treated with E-cadherin-Fc-coated beads were immunostained with anti-pY845 antibody, followed by quantifying the percentage of pY845-positive cells with and without bound beads (Kim et al., 2011). Images of serum-starved (−EGF) A341-D cells expressing either WT E-cadherin or the ENE mutant are in Fig. S5F. The field of view includes cells with and without beads. By separately analyzing the two populations, we determined the percentages of pY845-positive cells with beads and without beads (Fig. S5G). In serum-starved cells, bound E-cadherin-Fc beads had a negligible impact on the low percentage of pY845-positive cells. In cells without beads, the percentages of pY845-positive cells expressing WT E-cadherin, ENE or Δ525–585 were statistically similar (Fig. S5G). We then determined the percentage of pY845-positive cells treated with 3 nM EGF, with and without E-cadherin beads (Fig. S5F,G). E-cadherin-Fc bead attachment to WT E-cadherin-expressing cells reduced the percentage of pY845-positive cells significantly, in agreement with prior findings (Perrais et al., 2007). By contrast, bead binding did not affect the percentage of pY845-positive cells expressing either ENE or Δ525–585 mutants (Fig. S5G). The results support the hypothesis that E-cadherin ligation regulates EGFR activity through extracellular domain interactions and that E-cadherin association with EGFR is required.

### Disrupting E-cadherin–EGFR complexes increases EGF-dependent proliferation in dense cultures

To test whether E-cadherin suppresses EGFR signaling and proliferation through extracellular domain interactions, we compared proliferation (EdU uptake) in confluent monolayers of MCF 10A KO cells rescued with WT E-cadherin, Δ525–585 or Δ488–592 mutants. Comparisons were made across a range of EGF concentrations. These studies used MCF 10A cells, because they form well-defined, mature junctions and exhibit lower background EdU uptake than A431-D cells, when engineered to express WT E-cadherin.

**Fig. 5.**
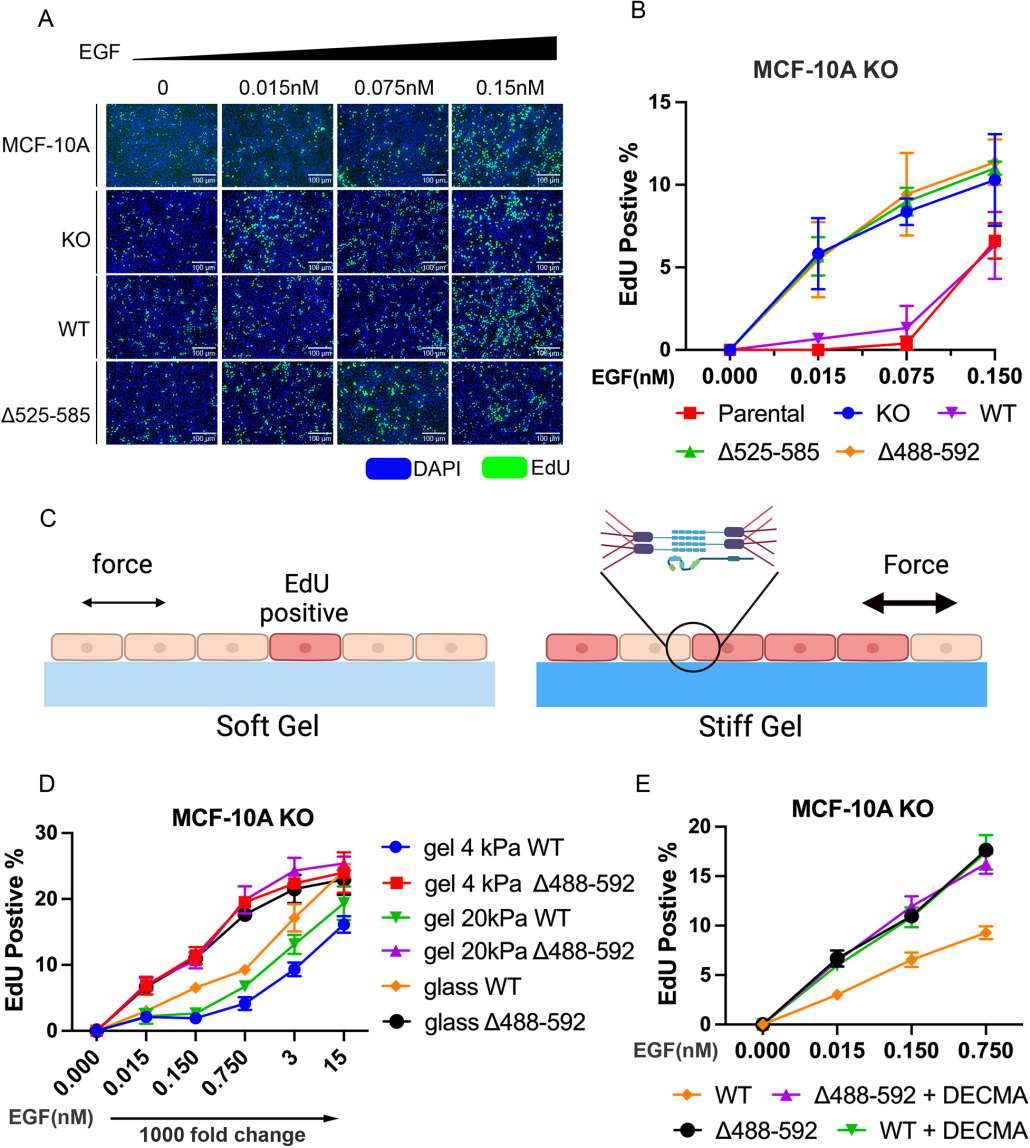
**Uncoupling E-cadherin–EGFR complexes disrupts tension-dependent proliferation in dense epithelial cultures.** (A) Representative fluorescence images of EdU (green) and DAPI (blue) in dense, confluent monolayers on fibronectin-coated glass, after treatment with EGF at the indicated concentrations. Data are shown for MCF 10A and MCF 10A KO cells, as well as for MCF 10A KO cells rescued with WT E-cadherin or the Δ525-585 mutant. (B) Percentage (%) EdU-positive cells versus EGF concentration (nM) measured under conditions shown in A, with MCF 10A parental cells, MCF 10A KO cells and MCF 10A KO cells rescued with WT E-cadherin or with Δ525–585 and Δ488–592 mutants. *N*=3 independent measurements. (C) Schematic of the experimental setup used to test the influence of E-cadherin–EGFR complexes on the tension-dependent regulation of proliferation in dense monolayers. EdU uptake was measured in confluent monolayers cultured on stiff (20 kPa) or soft (4 kPa), fibronectin-coated polyacrylamide gels. Substrate rigidity increases force across cell–cell junctions. (D) Percentage (%) EdU-positive cells versus EGF concentration, measured with MCF 10A KO cells rescued with WT E-cadherin or the Δ488–592 mutant. Cells were cultured on fibronectin-coated glass or polyacrylamide gels with Young's moduli of 4 and 20 kPa. Data were from *N*=3 independent measurements. (E) Comparison of the percentage (%) EdU-positive cells versus EGF concentration, with or without treatment with cadherin-blocking DECMA-1 antibody. Data are shown for confluent MCF 10A KO cells rescued with WT E-cadherin or the Δ488-592 mutant. Cells were on fibronectin coated glass. Data were from *N*=3 independent measurements. All data in B, D and E are represented as the mean±s.e.m.

Fig. 5A shows fluorescence images of DAPI and EdU-positive cells on fibronectin-coated glass at different EGF concentrations. The percentage of EdU-positive cells for each condition was determined by subtracting the baseline percentage measured in the absence of EGF. Quantitative comparisons of the percentage EdU-positive cells in the population under the different conditions are in Fig. 5B.

WT E-cadherin expression reduced EdU uptake compared to E-cadherin-null MCF 10A KO cells, at EGF concentrations up to 0.15 nM (Fig. 5B). At higher EGF concentrations, the EdU uptake by WT E-cadherin-expressing cells increased (see Fig. 5D). In contrast, with confluent monolayers expressing the Δ525–585 or Δ488–592 mutants, proliferation was significantly higher than that in WT-expressing cells and increased monotonically with increasing EGF.

### E-cadherin mutants alter tension-dependent proliferation on compliant substrates

Proliferation in confluent epithelial cultures reportedly increases with the rigidity of fibronectin-coated substrates (Kim and Asthagiri, 2011). Although integrins contribute to stiffness-dependent proliferation in isolated cells (Saxena et al., 2017), we predicted that cadherin force transduction is a major factor regulating rigidity-dependent proliferation in dense cultures in the presence of EGF. This is because increased integrin-dependent cell contractility on stiffer substrates increases intercellular tension (Liu et al., 2010; Maruthamuthu et al., 2011) (Fig. 5C), and E-cadherin tension increases EGFR signaling (Sullivan et al., 2022). If hetero-receptor complexes are intercellular mechano-switches, then higher junctional tension would be expected to increase proliferation in confluent monolayers that express WT E-cadherin, but not mutants that uncouple the complex. We tested this by comparing proliferation rates of cells on fibronectin-coated glass (∼70 GPa) versus on polyacrylamide (PA) gels with Young's moduli of 4 and 20 kPa (Fig. 5C). Here, substrate stiffness is used as a proxy for junctional tension (Liu et al., 2010; Maruthamuthu et al., 2011).

To estimate rigidity-dependent contributions to proliferation from integrins, we exploited findings that integrins activate EGFR independently of EGF (Saxena et al., 2017) but E-cadherin force transduction requires EGF (Sehgal et al., 2018; Sullivan et al., 2022). EdU uptake was thus measured in serum-starved confluent monolayers on the different substrates. MCF 10A KO cells expressing the Δ488–592 mutant or WT E-cadherin exhibited similar small statistically significant increases in EdU uptake with substrate stiffness (Fig. S5H). This ‘baseline’ was subtracted from measurements of cells on the same substrate, but in the presence of different concentrations of EGF. Although EGF might also increase contractility, the integrin contributions should be similar when comparing cells on identical substrates at the same EGF concentrations. Thus, differences in growth factor dependent proliferation would reflect the different cadherins expressed, rather than integrins.

Fig. 5D compares the baseline-subtracted percentages of EdU-positive cells in confluent monolayers on fibronectin-coated glass and on fibronectin-coated PA gels, measured at the indicated EGF concentrations. The data compare MCF 10A KO cells expressing the Δ488–592 mutant versus WT E-cadherin. There was greater EdU uptake by Δ488–592 versus WT E-cadherin-expressing cells at all EGF concentrations below ∼15 nM (Fig. 5D). Importantly, there are also clear rigidity-dependent differences in the proliferation of WT E-cadherin cells. At EGF concentrations above 0.015 nM, WT E-cadherin-expressing cells exhibited more EdU incorporation on glass versus on the softer PA gels. On both gels, proliferation was similarly low at EGF concentrations of <0.15 nM, but at concentrations >0.15 nM EGF, EdU uptake was higher in cells on 20 kPa versus 4 kPa gels.

In contrast to WT E-cadherin, the substrate stiffness had no influence on the EGF-dependent proliferation by Δ488–592-expressing cells at any EGF concentration (Fig. 5D). Because culture conditions are otherwise identical for both cell types, we attribute the rigidity-dependent EdU uptake by WT E-cadherin cells to the force-sensitive E-cadherin–EGFR complexes. Consistent with this view and other results of this study, complex disruption dysregulates cadherin mechanotransduction and rigidity-sensitive proliferation at low EGF concentrations.

To test whether the trends in EdU uptake depend on force across E-cadherin complexes, adhesion was disrupted with the E-cadherin blocking antibody DECMA-1. Fig. 5E compares EdU uptake by cells expressing WT E-cadherin versus Δ488–592 on fibronectin-coated glass. These measurements used glass because cell contractility and junctional tension should be highest on this rigid substrate. Without DECMA-1, the proliferation of Δ488–592 cells was higher than WT E-cadherin cells at all EGF concentrations used (Fig. 5E), and disrupting adhesions with DECMA-1 had no significant impact on proliferation. By contrast, with WT E-cadherin-expressing cells, DECMA-1 treatment increased proliferation to the same levels as mutant-expressing cells at all EGF concentrations. This result further supports the hypothesis that E-cadherin regulates cell proliferation through a force-sensitive complex with EGFR.

## DISCUSSION

This study investigated the basis of cooperation between EGFR and E-cadherin in intercellular force transduction. Prior fluorescence imaging and co-IP measurements have demonstrated that E-cadherin and EGFR receptors form hetero-trimeric complexes on live cells, independently of their cytoplasmic regions (Sullivan et al., 2022). Mechanically perturbing full-length, WT E-cadherin resulted in complex dissociation, EGFR activation, and downstream ERK/MAPK signaling (Sullivan et al., 2022). However, colocalization does not prove mechanical regulation, and these new findings now demonstrate that hetero-receptor complex formation is obligatory for E-cadherin mechanotransduction and cytoskeletal remodeling at stressed junctions.

There are different proposed models of E-cadherin crosstalk with EGFR, but these results show that force transduction requires receptor association. They further localized an essential E-cadherin region to the EC4 domain of the extracellular region. The loss of force transduction by cadherin mutants is not due to compromised adhesion, because the mutants disrupt EGFR co-immunoprecipitation but not E-cadherin adhesion. Although the receptors also reportedly interact indirectly through cytosolic proteins Merlin and NERF (also known as ELF1) (Curto et al., 2007), the cytoplasmic domain of E-cadherin did not detectably promote complex formation in A431-D cells. EGFR did not co-immunoprecipitate with either IL2R chimera, which both contain the E-cadherin cytodomain. Also, EGFR was depleted from junctions between A431-D and MCF 10A KO cells, when EC4 mutants were expressed, despite their intact cytoplasmic regions. These findings have potential implications for understanding disease progression and treatment. For example, several germline mutations in EC4 and adjacent sequences are linked to diffuse gastric cancer, including the point mutants used in this study (Berx et al., 1998; Hakkaart et al., 2019). Future work might further reveal how extracellular domain interactions cooperate with cytosolic binding partners to regulate EGFR signaling (Mendonsa et al., 2018).

Cadherin domain deletion mutants could disrupt hetero-receptor interactions, by misaligning *cis* interactions. This is not the case with the ENE chimera, which abolished EGFR co-IP and force transduction. On free cell membranes, two E-cadherin receptors reportedly bind one EGFR monomer (Sullivan et al., 2022), and mutations could indirectly affect EGFR binding, by disrupting *cis* E-cadherin dimers. Except for the IL2R chimeras, all mutants contain the *cis*-binding interface (Harrison et al., 2011) and a p120ctn (CTNND1)-binding region that supports constitutive *cis*-dimerization (Vu et al., 2021). Together these results support the hypothesis that force transduction requires E-cadherin–EGFR complexes.

We speculate that the EC4 domain harbors a force-sensitive hetero-receptor binding interface. The interaction could involve additional E-cadherin contacts given the large EGFR extracellular segment. Point mutations flanking EC4 attenuated but did not abolish hetero-receptor complex formation. An exon 8 deletion, as well as germline mutations T340A, P373L and A643V, for example, correlated with increased EGFR phosphorylation at cell–cell contacts (Mateus et al., 2007). Additionally, EGFR activation requires tension on homophilic *trans* E-cadherin bonds, such that tugging on cadherin with antibody or heterophilic ligand fails to activate EGFR or actin recruitment (Tabdili et al., 2012; Vu et al., 2025). This (cadherin) ligand selectivity might suggest that EGFR directly interacts with E-cadherin through a *cis* binding interface that senses the cadherin ligation status. We did not investigate EGFR mutants, because they could impact other functions, such as integrin signaling (Saxena et al., 2017), that also impinge on cadherin force transduction signatures.

**Fig. 6.**
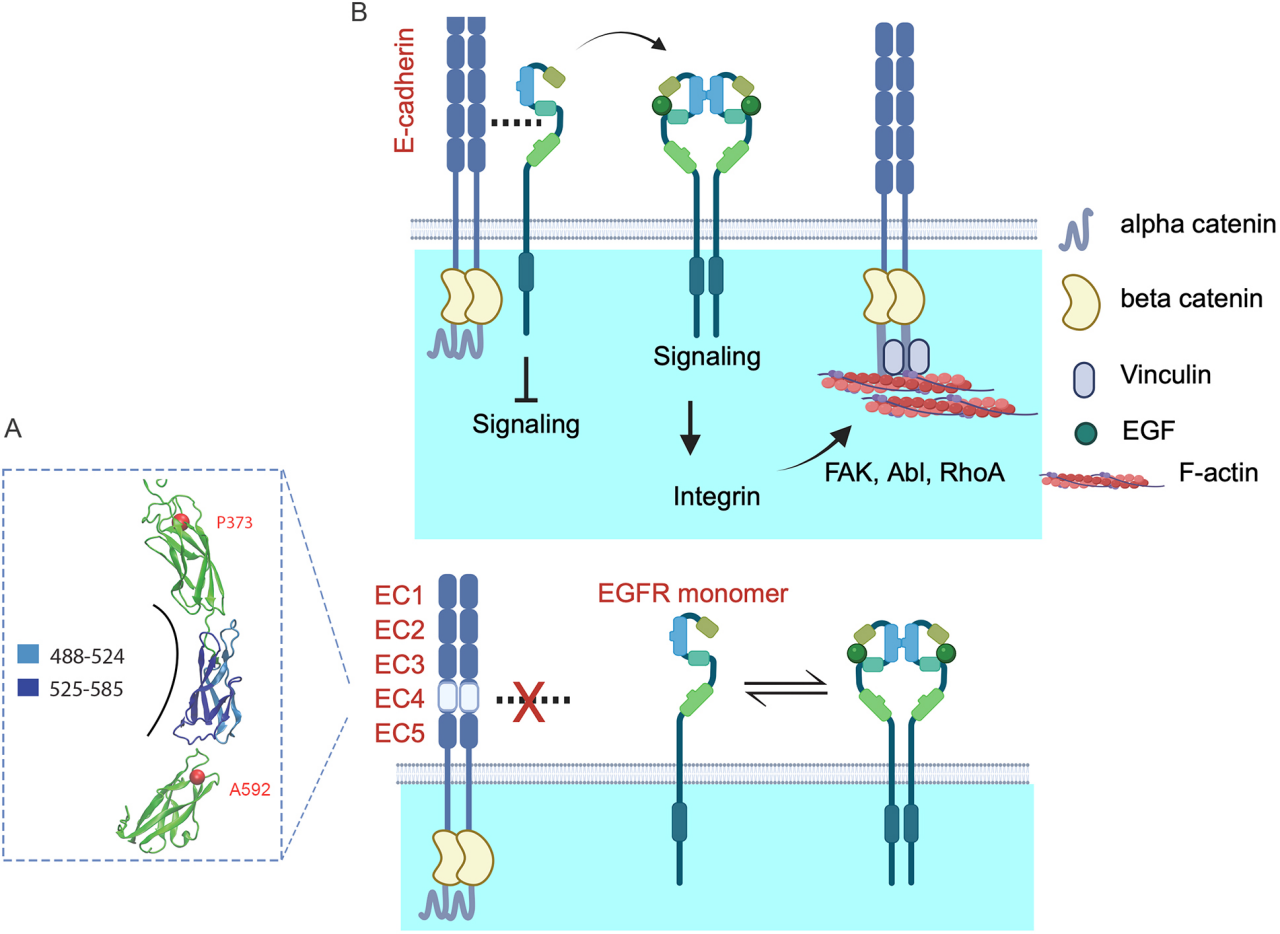
**Proposed E-cadherin association with EGFR and mechanically activated signaling at intercellular junctions.** (A) Structure of E-cadherin extracellular domains 2–5 showing the locations of EC4 (blue) and the locations of point mutants at P373 and A592 (red spheres). (B) Top, proposed EC4 mediated E-cadherin–EGFR association. Force on *trans* E-cadherin bonds induces hetero-receptor complex dissociation, which enables EGFR monomers to dimerize, bind EGF and signal. EGFR signaling potentiates vinculin recruitment and actin remodeling, via integrins, FAK, Abl kinase and RhoA. Bottom, mutating or deleting EC4 abolishes EGFR binding. EGFR is decoupled from tension on E-cadherin complexes, and this uncoupling eliminates the mechanical regulation of EGFR signaling and downstream cascades shown in B.

The ability of integrin activation, in conjunction with α-catenin unfurling, to rescue cell stiffening and actin recruitment, when E-cadherin and EGFR are uncoupled, confirms the role of integrins in cadherin force transduction cascades. Prior studies showed that integrins are activated downstream of cadherin tugging (Collins et al., 2012; Sehgal et al., 2018). The present results show that E-cadherin must associate with EGFR, to mechanically activate signaling to downstream integrins, which regulate vinculin and actin recruitment to stressed cadherin complexes (Kong et al., 2022). Integrin pre-activation alone did not rescue force transduction. However, integrin activation in conjunction with E-cadherin tension, which unfurls α-catenin, rescued actin recruitment and cell stiffening. These results confirm that integrins are key effectors of force-activated EGFR (Figs 4A, 6; Kong et al., 2022), thus linking cadherin-initiated EGFR signals to cell contractility and cytoskeletal reinforcement at E-cadherin junctions.

These results address long-standing questions regarding E-cadherin interactions with EGFR and the mechanical activation of signaling at epithelial junctions. They support proposed models of cadherin-mediated mechano-transduction cascades (Kong et al., 2022; Sehgal et al., 2018). α-catenin is required to both mechanically couple cadherin to actin and scaffold vinculin and actin, but mechanically regulated EGFR and integrin signaling are also crucial components that promote cell contractility and cytoskeletal reinforcement at intercellular junctions.

E-cadherin also mechanically regulates proliferation in dense epithelial cultures. A previous report (Kim and Asthagiri, 2011) hypothesized that the override of CIP upon matrix stiffening was due to cooperation between E-cadherin and the EGF receptor, but the underlying mechanism was not identified. In this study, the use of complex-disrupting E-cadherin mutants enabled us to show that the mechano-sensitive E-cadherin–EGFR complexes control the rigidity-dependent sensitization of proliferation to low EGF concentrations. These results also provide new insight into observations that mechanical strain in epithelia promotes cell cycle reentry through yes-associated-protein 1 (Yap1) and β-catenin (Benham-Pyle et al., 2015), whereas cytoskeletal tension at E-cadherin junctions affects the Hippo pathway through LATS1 and/or LATS2 recruitment (Ibar et al., 2018). The signals upstream from the nuclear accumulation of Yap1 in stretched epithelia were not identified previously. Our findings suggest that force-dependent EGFR activation by E-cadherin contributes to this behavior, through a mitogen (growth factor)-dependent pathway (Kim et al., 2011).

The disruption of WT E-cadherin adhesions (DECMA-1) increased proliferation to similar levels to those seen in confluent cultures expressing Δ488–592 (Fig. 5). The corresponding drop in bond tension did not reduce proliferation, as culturing cells on soft gels does, because *trans* cadherin ligation is also required for EGFR suppression (Perrais et al., 2007; Qian et al., 2004). Thus, both cadherin ligation and junctional tension regulate EGFR signaling, likely through a coupled mechanism. A plausible explanation is that *trans* cadherin ligation allosterically switches on *cis* EGFR binding. Then, as tension increases the dissociation of *trans* cadherin bonds (Bell, 1978), *cis* EGFR binding would switch off, to dissociate the complex and switch on signaling. However, demonstrating such structural changes is beyond the scope of this work.

The interplay between force-regulated E-cadherin–EGFR signaling and integrin activation also couples intercellular mechanotransduction to broader integrin signaling networks that regulate tissue functions. Future studies of the mechanical regulation of E-cadherin–EGFR complexes might also uncover mechanisms of disease progression and account for complex phenotypes observed in gastric cancers. More broadly, different cadherin subtypes interact with other receptor-tyrosine-kinases, raising the possibility that similar mechanisms regulate physiological processes in other tissue contexts (Vu et al., 2025).

## MATERIALS AND METHODS

### Reagents and antibodies

Fibronectin was from Millipore (Millipore FC-010). Hank's balanced salt solution (HBSS) with or without Ca^2+^ were from Corning (Corning, 21021CV, 2023CV), phosphate-buffered saline (PBS) was from Corning (21040CM). Tris-buffered saline with Tween 20 (TBST) was made with 20 mM Tris-base (Sigma, TRIS-RO), 150 mM NaCl (Sigma, S9888) and 0.1% Tween 20 (Thermo Fisher Scientific, T/4206/60). All restriction enzymes were from New England Biolabs (NEB).

Monoclonal, rat anti-EGFR antibody (1:1000; Cell Signaling Technology, 4267) was used for western blots of EGFR and for immunoprecipitation. Polyclonal, anti-phospho-EGFR (Tyr845) antibody (1:1000: Thermo Fisher Scientific, 44-784G) produced in rabbit was used to detect EGFR phosphorylation. Immunoblots of E-cadherin and actin used mouse monoclonal anti-E-cadherin (1:1000; Thermo Fisher Scientific, BDB610181) and anti-actin (1:1000; BD Biosciences, 612656). Immunoblots of GST for detecting active integrin used anti-GST antibody (1:1000; Santa Cruz Biotechnology, sc-138). Secondary antibodies anti-rabbit IgG horseradish peroxidase (HRP) (1:5000; Sigma, A0545) and anti-mouse IgG HRP (1:5000; Promega, W402B) were used to detect protein levels by chemiluminescence. The antibodies anti-integrin α5 (1:200; Santa Cruz Biotechnology, sc-13547), anti-fibronectin (16G3) (1:200; from Kenneth Yamada, NIH, Bethesda, MD, USA), and DECMA-1 (1:200; Millipore Sigma, MABT26) were used to block, respectively, α5 integrin, formation of new integrin adhesions, and E-cadherin adhesion. Flow cytometry measurements of E-cadherin expression used Alexa-Fluor-647-conjugated anti-E-cadherin antibody G-10 (1:200; Santa Cruz Biotechnology, sc-8426). Immunofluorescence imaging of vinculin, E-cadherin and actin used anti-vinculin rabbit antibody (1:200; Cell Signaling Technology, E1E9V), anti-E-cadherin (1:200; Invitrogen, HECD-1) and Phalloidin–Alexa-Fluor-647 (Invitrogen, A22287). Secondary antibody for immunofluorescence imaging used anti-mouse IgG FITC-conjugated antibody (1:250; Invitrogen, F-2761) and anti-rabbit IgG Alexa-Fluor-555-conjugated antibody (1:250; Invitrogen, A10523).

### Cell culture

A-431D cells (gift from Keith Johnson, University of Nebraska, Lincoln, USA) were maintained in Dulbecco's modified Eagle's medium (DMEM, 4.5 g/l glucose, Corning, 90013PB) supplemented with 10% (v/v) fetal bovine serum (FBS, Gibco), 1 mM sodium pyruvate, 1% (v/v) penicillin-streptomycin (Invitrogen, 15070-063). E-cadherin mutants were expressed in A431-D cells and selected with 800 μg/ml geneticin sulfate (GoldBio, G-418-1). Cells were further sorted based on YFP fluorescence intensity, using either the Bigfoot Spectral Cell Sorter (Thermo Fisher Scientific; Keck Center, University of Illinois, Urbana-Champaign, IL, USA) or BD FACS ARIA II (BD Bioscience; Keck Center). The YFP intensity from A431-D E-cadherin-expressing cells were used to set the sorting gate, in order to obtain cells with similar expression levels of all E-cadherin mutants. The engineered cells were re-sorted occasionally, to compensate for clonal drift. MCF-10A and MCF-10A CDH1^−/−^ cells (gift from Barry Gumbiner) were maintained in DEME/F12 medium (Invitrogen, 11330-032) with 5% horse serum (Invitrogen, 16050-122), 20 ng/ml EGF (Peprotech, AF-100-15-500UG), 0.5 mg/ml hydrocortisone (Sigma, H0888), 100 ng/ml cholera toxin (Sigma, C8052) and 10 μg/ml insulin (Sigma, I3536). The parental and engineered A431-D cells were routinely tested for E-cadherin protein expression through western blotting or flow cytometry. The MDCK KO and rescued cells were similarly tested for E-cadherin expression, and we also visually inspected cell morphologies. Cells were tested for mycoplasma contamination [Tumor Engineering and Phenotyping (TEP) Shared Resource, UIUC].

### Primers

Primers used were: D488-F: 5′-TTTGTGCCTATACCAGAACCTCGAACTATATTCTTCTGTG-3′; D488-R: 5′-GTTCTGGTATAGGCACAAAGATGGGGGCTTC-3′; D525-F: 5′-CATATCGCTCTGATGTGAATGACAACGC-3′; D525-R: 5′-ACATCAGAGCGATATGTTATTTTCTGTTCCATAAATG-3′; A592T-F: 5′-TGACAACACACCCATA CCAGAACCTCGAACTATATTCTT-3′; A592T-R: 5′-TATGGGTGTGTTGTCATTCACATCAGACAGGATCA-3′; P373L-F: 5′-ATCCTCTGATCTTCAATCCCACCACGTAC-3′; P373L-R: 5′-AAGATCAGAGGATTATCGTTGGTGTCAGTGAC-3′; IL2R-KpnI-F: 5′-GGGTACCGAGGATCTTTGTGAAGGAACCTTAC-3′; IL2R-KpnI-R: 5′-TCCTCGGTACCCCTAGTGGTCCTCGCC-3′; IL2R-NheI-F: 5′-GCCGCCGCTAGCTTGGCGGCTG-3′;

IL2R-AgeI-R: 5′-GCCGCCACCGGTTGTTGTGACGAGGCAGG-3′; pbabe-ApaI-F: 5′-GCGCGGGGCCCATGACCGAGTACAAGCCCA-3′; pbabe-HindIII-R: 5′-GCCGGTAAGCTTTTTGCAAAAGCCTAG-3′; E-cadherin-HindIII-F: 5′-CCCAAGCTTATGGGTCCTTGGAGCCG-3′; and E-cadherin-ApI-R: 5′: 5′-GCGCGGGGCCCTCAGTCGTCCTCGCCG-3′.

### Protein and cell engineering

Human E-cadherin mutants with C-terminal YFP tags were engineered to disrupt *cis* interactions with EGFR but not *trans* cadherin binding. To test the role of the E-cadherin extracellular and transmembrane domains, the extracellular region was exchanged with the extracellular region of the interleukin 2 receptor (IL2R TMIC), or both extracellular and transmembrane domains were exchanged with the similar IL2R region (IL2R IC). The IC chimera consists of IL2R [amino acids (aa) Met^1^–Gln^262^] and E-cadherin [amino acids (aa) Leu^731^–Asp^882^] (Gottardi et al., 2001). A KpnI cutting site was engineered into the C-terminal end of the IC chimera and double digested with NheI and KpnI to harvest the IC sequence, which then was inserted into the pcDNA3.1-YFP vector (gift from Kalina Hristova, Johns Hopkins University, USA) with the same digestion site. The TMIC chimera was cloned by amplifying IL2R (aa Met^1^–Thr^217^) by PCR, with the forward primer having 5′-NheI and the reverse primer having AgeI-5′ restriction cutting sites. Then, the sequence was double digested to replace E-cadherin (Met^1^–Gly^690^) in pcDNA-E-cadherin–YFP to obtain the TMIC chimera [IL2R Thr^217^-E-cadherin (Gly^690^–Asp^882^))].

We also generated a chimera in which EC4 of E-cadherin was exchanged with the EC4 domain of N-cadherin (ENE) (Kim et al., 2000). To generate the ENE chimera, the partial sequence of EC4 (aa Tyr^523^–LIe^594^) of E-cadherin was exchanged with a structurally similar region from N-cadherin (aa Thr^535^–Gln^604^). The ENE sequence (aa Met^1^–Gly^690^) was flanked with NheI and AgeI (intrinsic cutting site resides at E-cadherin aa Gly^690^) from the original plasmid and subcloned to replace aa Met^1^–Gly^690^ of pcDNA3.1-E-cadherin-YFP.

The Δ488–592 and Δ525–585 proteins were generated by one-step QuikChange as described previously (Liu and Naismith, 2008). Briefly, designed primers with a flanking overlap sequence were used to amplify the plasmid lacking the designated deletion sequence. In the second phase, the primers ligated the amplified plasmid with the overlapping sequence, to delete the large fragment.

P373L and A592T were two HDGC germline missense mutations (Bremm et al., 2008; Petrova et al., 2016). A one-step QuikChange was used to make single nucleotide replacements (P373L, CCT-CTG-ATC>CCT-CCGATC, A592T: AAC-ACA-CCC>AAC-GCC-CCC). Forward and reverse primer sequences are given above. All sequences were verified by DNA sequencing in a 3730xl DNA analyzer (Applied Biosystems, CoreLims at the Roy J. Carver Biotechonology Center, University of Illinois, Urbana-Champaign, IL, USA)

WT E-cadherin and all mutants were stably expressed in A431-D cells and selected for similar cadherin expression, by quantitative flow cytometry. WT E-cadherin and mutants Δ488–592 and Δ525–585 were also stably expressed in an MCF 10A line in which E-cadherin was knocked out using CRISPR (gift from Barry Gumbiner). The latter E-cadherin KO cells were engineered to express WT cadherin or either mutant using lentivirus, and cells were selected for similar expression levels as the parental cells.

Recombinant E-cadherin ectodomains C-terminally tagged with the Fc domain of human IgG (E-cadherin-Fc) was produced from HEK293T cells engineered to stably express and secrete the protein (Prakasam et al., 2006).

### Viral infection

The E-cadherin mutants were cloned into the retroviral expression plasmid pBabe-puro (Addgene #1764, deposited by Hartmut Land, Jay Morgenstern and Bob Weinberg,). WT E-cadherin and E-cadherin mutant sequences were amplified from the pcDNA3.1 vector, using primers with flanking restriction cutting sites HindIII on the forward primer and ApaI on the reverse primer (see above). The pBabe-puro vector was amplified using primers with HindIII on the reverse primer and ApaI on the forward primer (see above). The E-cadherin PCR products were double digested with HindIII and ApaI and then ligated into the pBabe vector to generate pBabe encoding WT E-cadherin–YFP or the E-cadherin mutants. Next 8 µg of each expression plasmid was transfected into Phoenix-AMPHO retrovirus producer cells (ATCC, CRL-3213) in 10 cm dishes, using the manufacturer recommended Lipofectamine 2000 protocol (Invitrogen, 11668019). The virus-harvesting protocol was adapted from a published protocol describing the AMPHO system (Swift et al., 2001). Briefly, at 24 h post transfection, the medium was removed and changed with 5 ml DMEM with 10% FBS. At 48 h post-transfection, 5 ml of the virus-containing supernatants were collected and filtered through a 0.45 μm filter, to remove cell debris. Then, 1 ml of virus supernatant was added to confluent MCF-10A KO cells on a six-well plate, in the presence of 8 µg/ml polybrene (Santa Cruz Biotechnology, sc-134220), for an overnight infection at 37°C. At 24 h post infection, DMEM/F12 medium (Invitrogen) containing 10 µg/ml puromycin (Invivogen, ant-pr-5) was used to select the infected population.

Cells were sorted to further select cells for similar protein expression levels. Briefly, confluent monolayers of cells were harvested from the six-well plates by treatment with TrypLE (Gibco, 12604) in medium containing 1 mM Ca^2+^. The harvested cells were stained for 45 min with G-10 anti-E-cadherin antibody conjugated with Alexa-Fluor-647 (Santa Cruz, sc-8426) in 200 μl HBSS with 1% BSA. After two washes with PBS, cells were sorted with the Bigfoot cell sorter (Keck Center). MCF-10A parental cells were used as the positive control for E-cadherin expression and to set the gate for sorting cells with similar protein expression across all mutants. The engineered cells were re-sorted occasionally, to compensate for clonal drift.

### Quantification of cadherin surface densities

Flow cytometry measurements quantified the density of cadherins on cell surfaces (cadherin molecules/µm^2^). Subconfluent cells were harvested under protease-free conditions. Cells were treated with 5 mM EDTA in PBS for 15 min, followed by gentle agitation to shear cells from the plate. Two washes with PBS were performed and the cell number was then counted. Cells expressing E-cadherins were labeled with 1:100 dilution of the Alexa-Fluor-647 conjugated anti-E-cadherin antibody G-10 (Santa Cruz Biotechnology, sc-8426) in 200 µl of HBSS for 45 min, followed by two HBSS washes. The fluorescence intensity of the labeled cells was measured with an LSR II flow cytometer (BD Biosciences; Keck Center) or the Symphony A1 (BD Biosciences; Keck Center). The calibration curve used to relate the fluorescence intensity per cell to the cadherin surface density was generated with calibrated Alexa-Fluor-647-labeled standard beads (Bangs Laboratories, Fishers, IN, USA).

### Co-immunoprecipitation assays

Co-IP measurements of E-cadherin and EGFR were conducted using standard procedures (Sullivan et al., 2022). Confluent monolayers were washed twice in ice-cold HBSS and lysed on ice with 1% Triton X-100 lysis buffer (20 mM Tris HCl pH7.4, 137 mM NaCl and 1.2 mM CaCl_2_) supplemented with protease and phosphatase inhibitor cocktails (Thermo Fisher Scientific, PIA32955; Sigma, P0001-1ML) for 30 min at 4°C under gentle rocking. Meanwhile, protein-G conjugated magnetic beads (Bio-Rad, 1614023) were incubated with a 1:100 dilution of monoclonal anti-EGFR antibody (Cell Signaling Technology, D38B1) in HBSS for 30 min at 4°C. Cell lysates were then centrifuged for 10 min at 14,000 ***g*** to remove cellular debris, and the supernatant was collected. Protein-G beads were washed three times with PBST (PBS with 0.1% Tween 20) to remove excess antibody. After washes, supernatants were discarded, and beads were collected. The lysate supernatant was then added to magnetic beads and incubated at 4°C for 1 h. Before eluting the protein, the beads were washed three times with PBST. Next, 20 µl of Laemmli buffer (Bio-Rad, 1610737) was added to beads and the samples were boiled for 10 min to strip proteins from the beads. Western blots were used to identify the protein content in the resulting samples. Details of the blot analyses are described below.

### SDS-PAGE and western blot analysis

Proteins were separated on a 4–20% gradient SDS-PAGE gel (Bio-Rad, 4561096) then transferred onto a PVDF membrane (Thermo Fisher Scientific). For co-IP samples, the membrane was first incubated with anti-E-cadherin (BD Bioscience, 610181) in 5% (w/v) bovine serum albumin (BSA)-TBST (10 mM Tris-HCl pH 7.4, 150 mM NaCl, 0.1% Tween-20) overnight at 4°C using 1:1000 dilution. The blot was washed five times with TBST, followed by the addition of horseradish peroxidase (HRP)-conjugated anti-mouse IgG (1:5000, Promega, W4021) secondary antibody in TBST. After a 90 min incubation at room temperature, the blot was washed three times in TBST for 5 min a wash and then the membrane was imaged with chemiluminescence based ECL substrate and iBright system to capture grayscale intensities. Following imaging, the blot was stripped with stripping buffer (Thermo Fisher Scientific, 21059) for 45 min at room temperature. The membrane was washed twice in TBST for 5 min before re-probing overnight with anti-EGFR (Cell Signaling Technology, D38B1). After the overnight incubation, the membrane was washed three times with TBST and then incubated with HRP conjugated anti-rabbit IgG (1:5000, Sigma) for 90 min, followed by three washes with TBST and imaging. For the primary antibody incubation, a 1:1000 dilution was used for all antibodies, unless otherwise indicated.

For co-IP quantification, the grayscale intensities of E-cadherin and EGFR bands on the blotted membranes were quantified using the Gels plugin in ImageJ. Only raw images were used for quantification. For representative images shown in the figures, minimal contrast enhancement was applied solely to improve visualization. Any enhancements were applied uniformly across all images, including control lanes. All western blot analyses followed the above procedures.

### Shear flow adhesion assay

Glass capillary tubes (1.0 mm internal diameter) were coated with 50 µg/ml E-cadherin-Fc in HBSS overnight at 4°C. The capillary was washed three times with HBSS by gentle pipetting and subsequently blocked with 10 mg/ml BSA in HBSS for 2 h. at room temperature. Cells were pre-seeded sparsely at 24 h before the measurements. Before harvesting, cells were washed twice with PBS to remove any proteases. Then cells were harvested with PBS containing 5 mM EDTA for 5 min, followed by two washes and a final resuspension in Ca^2+^-containing HBSS to obtain 2×10^5^ cells per ml. For negative control experiments, cells were washed twice in HBSS with Ca^2+^, but the cells were resuspended in HBSS without Ca^2+^.

Cells were initially injected into the cadherin (or antibody)-coated glass capillary and mounted on an upright Olympus microscope BX-60. The capillaries were connected to a 60 ml syringe attached to a syringe pump (Harvard instrument, S. Natick, MA, USA). A 0.5 ml/min flow was used to draw cells further to the center of the capillary. After a 20 min incubation at room temperature, the number of cells in a 20× objective field was counted. Flow was then initiated for which the rate was increased every 30 s, and the remaining number of cells was counted at the end of each time interval. A range of 0–16 ml/min flow was used to assess the relative cell adhesion. For assessing the adhesion of the IC mutant, 50 µg/ml anti-IL2R antibody (Abcam, ab-9496) was used to coat the capillary. Data are reported as percentage of cell remaining, relative to the initial time point as 100%, versus wall shear stress (WSS) calculated with flow rates. The wall shear stress at which 50% of the cells detached, WSS_50_, was used to compare the relative cell adhesion. The latter was determined from degree 2 polynomial fits of the data.

### Magnetic twisting cytometry

MTC was used to mechanically perturb E-cadherin receptors as described previously (Muhamed et al., 2016). Briefly, ferromagnetic beads (4.82 µm diameter, Spherotech, Lake Forest, IL, usA) were activated by treatment with 10 mg/ml 1-ethyl-3-(3-dimethylaminopropyl)-carbodiimide hydrochloride (EDC, Sigma-Aldrich) and N-hydroxysuccinimide (NHS, Thermo Fisher Scientific) in 300 µl MES buffer (50 mM MES, 100 mM NaCl, pH 5.0) for 15 min at room temperature. Activated beads were then incubated with 10 µg Fc-E-cadherin EC1–EC5 in HBSS for 2 h in 4°C with 600 rpm shaking on an orbital shaker. After protein immobilization, beads were washed twice to remove unbound Fc-Ecad.

The E-cadherin-Fc, anti-IL2R antibody or PLL control modified beads were incubated with monolayers of cells for 40 min at 37°C, under 5% CO_2_. The ferromagnetic beads were first aligned with a short field pulse of 1 Tesla, then twisted with an orthogonal, oscillating magnetic field of 60 Gauss at 0.33 Hz for 120 s. The resulting torque drives bead displacements and generates a modulated shear stress on E-cadherin bonds. The bead displacements were tracked with an inverted microscope (Zeiss Axiovert 100M) with a 20× objective. The change of displacement amplitude was a signature of force transduction and a reflection of change in elastic modulus of the bead–cell junction, which can be calculated as described previously (le Duc et al., 2010). Data are reported in terms of the percent change of bead-cell stiffness, relative to the initial elastic modulus, as a function of twisting time. The changes in stiffness follow a log normal distribution, from which we calculate the mean and s.e.m. For each cell type, at least 150 beads were analyzed per condition at one bead/cell, to determine the mean±s.e.m. at each time point.

### Integrin activation

The GST-tagged fragment of fibronectin domains 9–11 (GST–Fn_9-11_) used to probe activated α5β1 integrin (Coon et al., 2015) after bead pulls or Mn^2+^ treatment, by immunoblotting with anti-GST antibody, as described previously (Kong et al., 2022). Integrins were pre-activated with 0.5 mM MnCl_2_ for 20 min, before MTC measurements (Muhamed et al., 2016). The 20 min incubation produces more potent integrin activation than shorter incubation times (Coon et al., 2015). Cells were incubated with E-cadherin-coated beads for 20 min, and then the medium was changed to DMEM with 0.5 mM MnCl_2_. Bead twisting was initiated 20 min after Mn^2+^ addition. Cells were then fixed and immunostained, prior to imaging vinculin and actin at the E-cadherin-Fc coated beads. Western blots with anti-GST antibody were used to quantify integrin activation. Mechanically perturbed cells in MTC experiments were washed twice in HBSS and incubated with 20 µg/ml GST–Fn_9-11_ at 37°C for 25 min. Cells were lysed on the plate with Laemmli sample buffer (Bio-Rad, 1610373), then analyzed by SDS-PAGE and immunoblotting with an anti-GST antibody (Santa Cruz Biotechnology, sc-138).

### Confocal and super resolution imaging

The force-activated recruitment of actin and vinculin recruitment to stressed cadherin receptors were assessed by confocal immunofluorescence imaging. In MTC measurements, cells were fixed in 4% PFA for 10 min. After two washes in PBS, the fixed samples were permeabilized with 0.1% Triton X-100 in PBS for 10 min, followed by washing three times in PBS. Then, samples were blocked with 1% BSA in PBS for another 30 mins at room temperature. Vinculin was stained overnight with rabbit anti-vinculin antibody (Cell Signaling Technology, E1E9V) at a 1:200 dilution in PBS, followed by an incubation at room temperature with goat-anti-rabbit IgG Alexa-Fluor-555-conjugated antibody (Invitrogen, A10523) at 1:200 dilution in PBS for 90 min. Actin was stained by incubating samples with Phalloidin–Alexa- Fluor-647 (Invitrogen, A22287) at a dilution of 1:400 in PBS, at room temperature for 90 min. Confocal images were acquired with a LSM 700 with a 63×/0.75 NA oil immersion objective. Obtained images were quantified with ImageJ. Briefly, the mean background-subtracted fluorescence intensity (MFI) of either actin or vinculin around beads was quantified in a region of interest (ROI) defined by a band within 1 µm of the periphery of the bead. The background was determined by a nearby region outside of the cell, using same ROI mask. After background subtraction, the MFI determined for samples with or without load. At least 100 beads were analyzed for each condition. Statistical significance was determined by a two-tailed unpaired Student's *t*-test. *P*<0.05 was taken as statistically significant. Data are reported as mean±s.e.m. unless otherwise indicated.

Super resolution images were acquired with a Zeiss LSM 900 confocal microscope with an Airyscan2 module. The theoretical *x*/*y* resolution for the microscope is 120 nm with a pixel size of ∼40 nm (Huff, 2015). To stain E-cadherin and EGFR, cells were fixed with 2% PFA and permeabilized with 0.1% Triton X-100 for 5 min. Then, cells were incubated over-night at 4°C with mouse-anti-E-cadherin (Invitrogen, HECD-1) and rabbit-anti-EGFR (Cell Signaling Technology, D38B1) at 1:200 dilution in 1% BSA in PBS. After primary antibody incubation, cells were washed three times in PBS, followed by incubation with 1:500 anti-mouse-IgG–FITC (Invitrogen, F-2761) and goat-anti-rabbit IgG Alexa-Fluor-555-conjugated antibody (Invitrogen, A10523) in 1% BSA in PBS for 90 min at 37°C. Images were then acquired in Airyscan super resolution (SR) mode with the LSM 900 microscope. The ImageJ plug-in Coloc2 was used to quantify E-cadherin and EGFR colocalization, based on the Pearson's correlation coefficient (Dunn et al., 2011). Image handling for analysis follows published guidelines (Dunn et al., 2011). In brief, to quantify the junctional colocalization of E-cadherin and EGFR, a box-form ROI was set to enclose bright continuous E-cadherin fluorescence at intercellular junctions to identify stable cadherin intercellular junctions. Then, colocalization analysis was performed on the ROI with Costes threshold regression and a default point spread function (PSF) equal to 3.0, to minimize the contribution of background to false positive colocalization discovery.

### EdU uptake assays

Cell proliferation was assessed by EdU (Vectoc lab, CCT-1149) uptake. MCF 10A and MCF 10A KO (E-cadherin^−/−^) cells expressing WT E-cadherin or mutants were seeded on fibronectin-coated (10 µg/ml) 13 mm glass bottom dishes or on glass slides with fibronectin-coated polyacrylamide gels with Young's moduli of 4 or 20 kPa.

At 48 h post seeding on glass, cells were serum-starved for 24 h in serum-free DMEM/F12, after which the medium was changed to either serum-free DMEM/F12 with 10 µM EdU or with DMEM/F12 with 3 mM EGF and 10 µM EdU. After 18 h, the cells were washed with PBS and fixed. Next, the Cy5 fluorophore was coupled to EdU (Salic and Mitchison, 2008). Cells were then washed and mounted (Invitrogen, S36972) for imaging (Axiovert 200M). Briefly, cells were treated with 10 mM Azide-cy5 (Vector lab, AZ-118), 100 mM sodium ascorbate (Sigma, A4034-100G), 2 mM copper sulfate (Thermo Fisher Scientific, C493-500), and 0.2 µg/ml DAPI (Invitrogen, D1306) in 250 µl PBS for 90 min at room temperature. Cells were then washed twice and mounted (Invitrogen, S36972) for imaging with an Axiovert 200 M microscope. DAPI and Cy5 images were collected and analyzed using ImageJ. Briefly, the ‘auto-threshold’ plugin was used to remove background to visualize the nuclear shape. Next, images were converted into binary images with the ‘Watershed’ plugin. The number of nuclei represents the cell number, which were counted with the ‘analyze particles’ plugin. Proliferating cells were reported as the percentage of dividing, Cy5-positive nuclei relative to the total number of nuclei, determined from DAPI images.

Polyacrylamide hydrogels were prepared as described previously (Andresen Eguiluz et al., 2017; Tse and Engler, 2010). Glass coverslips (Cell E&G, GBD00002-200) were cleaned by soaking in 0.1 M NaOH overnight at room temperature, after which they were rinsed with ultrapure water and then air dried. The clean slides were then incubated with neat 3-aminopropyltrimethoxysiliane (APTMS) for 6 min, followed by three ethanol washes. Then, glasses were treated with 0.5% glutaraldehyde for 30 min at room temperature. Gels with Young's moduli of 4 or 20 kPa were prepared, by varying the acrylamide to bis-acrylamide (Bio-Rad) ratios (4 kPa: 5% acrylamide to 0.15% bis-acrylamide; 40 kPa: 8% acrylamide to 0.26% bis-acrylamide). The solution was degassed, followed by the initiation of gel polymerization by adding ammonium persulfate solution (APS, Bio-Rad) and tetramethyl-ethylenediamine (TEMED, Bio-Rad) to a final concentration of 0.05 w/v% (APS) and 0.05% (TEMED). The solution was layered onto the glass slide, and a perfluorosilane-coated glass cover slip was placed on top of the gel mixture. After polymerization was complete, the slide was removed to create a flat surface.

The gels were next covalently modified with fibronectin, using Sulfo-SANPAH (Thermo Fisher Scientific). A freshly prepared 1 mg/ml solution of Sulfo-SANPAH was added to the gels, which were exposed to UV light (360 nm, 4.4 mW/cm^2^, Spectroline XX-15A benchtop UV lamp) for 30 min. After activation, 0.05 mg/ml of Fibronectin (Fn, EMD Millipore) in PBS was incubated overnight with the Sulfo-SANPAH-treated gels at 4°C. Protein modification does not affect the gel stiffness (Tse and Engler, 2010). Before cell seeding, the gels were rinsed with PBS and then UV sterilized for at least 30 min. Cells were seeded at high density and grown to confluence on the gels (7 days) before experiments. After cells achieved confluence, they were serum starved for 24 h and then treated with different concentrations of EGF and 10 µM EdU. EGF concentrations from 0.015 nM to 15 nM were prepared by serial dilution of a 200µM stock solution in PBS. The low concentrations are consistent with those used in other studies (Kim and Asthagiri, 2011; Maruthamuthu et al., 2011). In addition, the estimated cell density-dependent dissociation constants (*K*_d_) between EGFR and EGF were estimated to be ∼0.12 nM and ∼6.5 nM, with the higher *K*_d_ population increasing with degree of confluence (Qian et al., 2004).

At 18 h post EGF and EdU treatment, cells were fixed with 4% PFA, followed by DAPI staining and use of a click reaction to couple Cy5 fluorophores to EdU. The number of EdU-positive cells relative to the total number of cells (DAPI) was quantified from fluorescence images.

### Use of artificial intelligence tools

ChatGPT was used to assist with proofreading during the preparation of the first draft of the manuscript.

## Supplementary Material

10.1242/joces.264350_sup1 Supplementary information
