# Supplementary material for: Epidermal growth factor receptor is an essential component in E-cadherin force transduction complexes
Source: J Cell Sci. 2025 Nov 13;138(21):jcs264350. doi: 10.1242/jcs.264350 (PMC12669962; doi:10.1242/jcs.264350)
Supplement: Supplementary information [file joces-138-264350-s1.pdf]

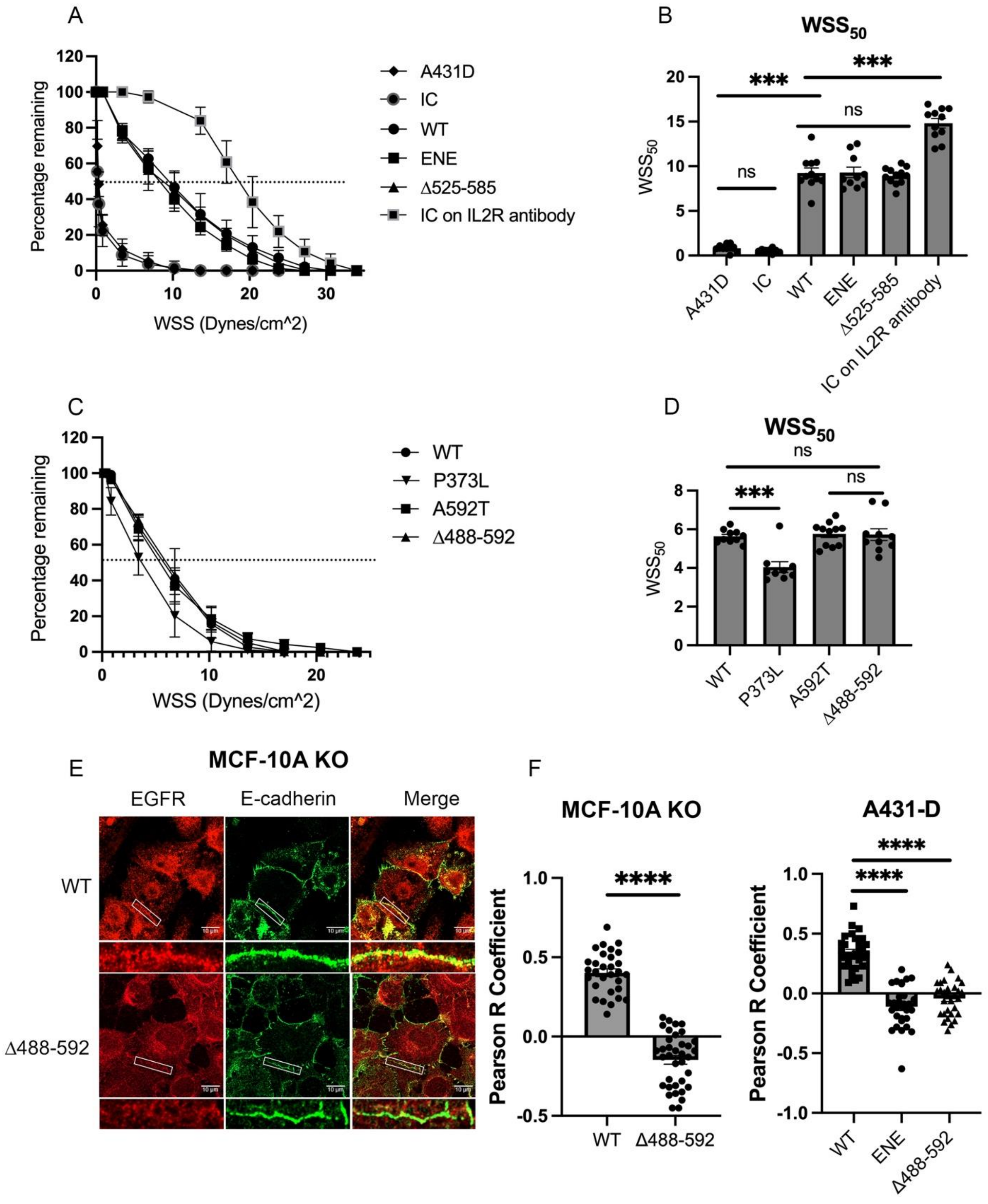

Figure S1

Journal of Cell Science • Supplementary information

Fig. S1. (Supplement to Fig. 1). Relative adhesion strength of E-cadherin mutants and EGFR co-localization. A) Percentage of remaining cells in E-cad-Fc coated capillaries, as a function of the wall shear stress (WSS, dynes per cm^2). Data compare the relative adhesion of A431-D cells expressing the IL2R-IC chimera, WT E-cadherin, ENE, or Δ525-585 to E-cad-Fc coated capillaries. In measurements with IL2R-IC expressing cells, capillaries were coated with anti-IL2R antibody. Controls used A431-D cells, which lack endogenous E-cadherin expression. The dashed line indicates 50% retention (WSS_{50}). B) Bar graph of the wall shear stress at which 50% of the cells remain, WSS_{50}. Values were determined from plots in part A. N ≥ 6 independent experiments. The data are represented as the mean ± s.e.m. C) Percentage of remaining cells as a function of the wall shear stress (WSS, dynes per cm^2). Data compare the relative adhesion of A431-D cells expressing WT, P373L, A592T, or Δ488-592 bound to E-cad-Fc coated capillaries. The dashed line indicates 50% retention (WSS_{50}). D) Bar graph of WSS_{50} values determined from plots in part C. N ≥ 6 independent experiments. All data are represented as the mean ± s.e.m. E) Representative super resolution fluorescence images of WT or ∆488-592 E-cadherin variants (green) and EGFR (red) at junctions between MCF-10 KO cells reconstituted with WT or Δ488-592 E-cadherin. Regions of Interest are indicated by white boxes around the E-cadherin junctions. The scale bar is 10 microns. F) Bar graph of the Pearson's correlation coefficient for the co-localization of EGFR with WT, ENE, or ∆488-592 E-cadherin variants in A41-D cells or with WT and Δ488-592 E-cadherins in MCF-10A KO cells. Colocalization quantification was represented with calculated Pearson's correlation coefficient. Number of junctions, n = 30, N = 3 independent experiments. **** p<0.0001.

Journal of Cell Science • Supplementary information

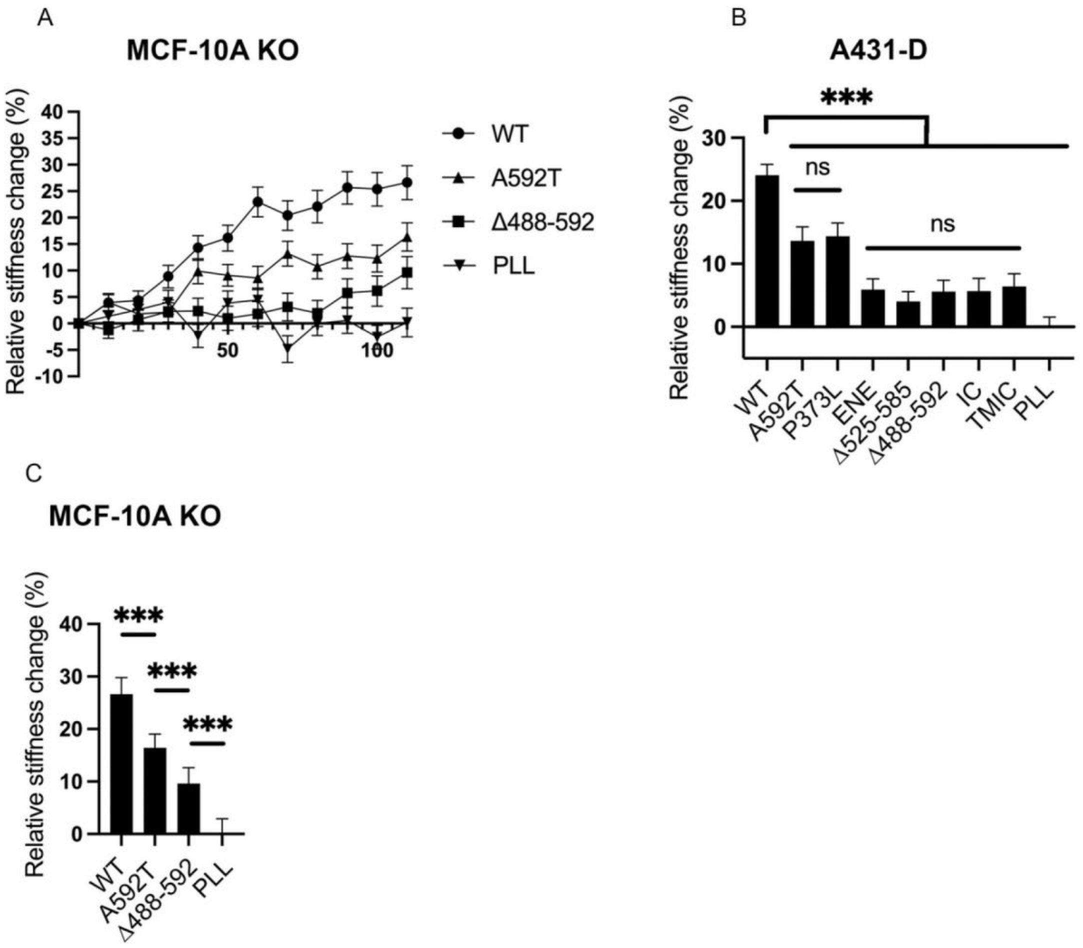

Fig. S2. (Supplement to Fig. 2). MTC measurements of the adaptive stiffening of cells reconstituted with E-cadherin variants. A) Percentage stiffness change measured with MCF-10A KO cells expressing WT E-cadherin, A592T, and Δ488-592. Negative controls used PLL coated beads. B) Final stiffness changes after 2min of bead twisting obtained with A431-D cells expressing the indicated mutants. n > 150 beads, N = 3. C) Bar graph of the final % stiffness change, from data in part A. Data show are the mean ± s.e.m. **** p<0.0001. n > 150 beads, N = 3 independent experiments.

Journal of Cell Science • Supplementary information

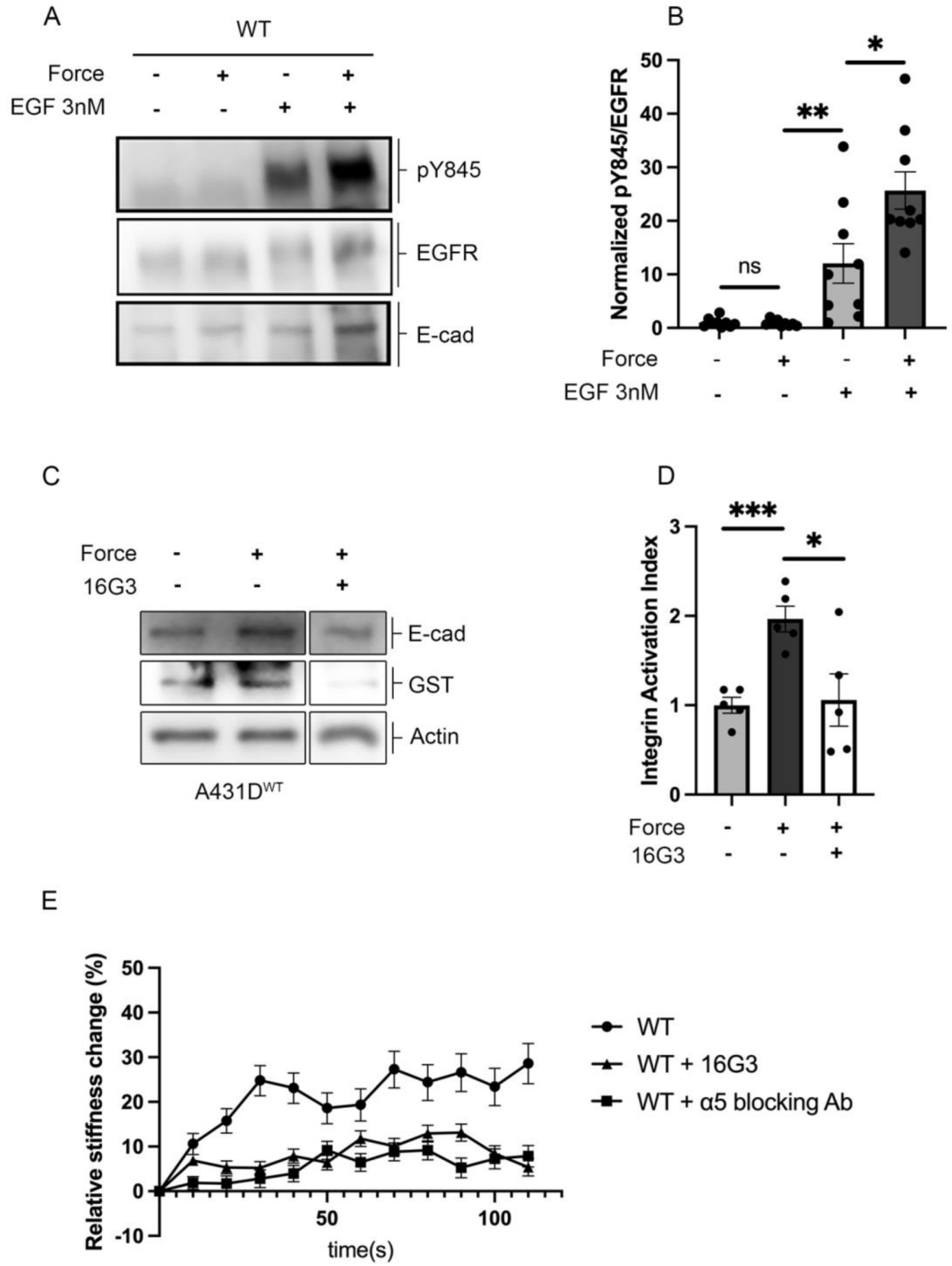

Fig. S3. (Supplement to Fig. 3). A) Representative Western blot of pY845, EGFR, and E-cad measured after stimulating E-cadherin receptors with E-cad-Fc modified beads. Measurements were done with or without force loading, in the presence or absence of 3nM EGF. B) Graph of quantified Western blots from part A. Individual pY845/EGFR ratios for each condition are by the data obtained with cells treated with

Journal of Cell Science • Supplementary information

3nM EGF without force loading. Data show the mean ± sem. N = 7 independent experiments. ** $p < 0.005$, * $p < 0.05$. C) Representative Western blot of GST-FNIII$_{9\text{-}11}$ incorporation (integrin activation index), with or without cadherin loading and with or without treatment with the fibronectin blocking antibody, 16G3. D) Bar graph of the quantified, relative integrin activation index, normalized to unperturbed cells. Data show the mean ± sem. N = 5 independent experiments. ** $p < 0.005$, * $p < 0.05$. E) Relative stiffness change (%) measured with A431-D cells expressing WT E-cadherin. Cells were treated with 16G3 or anti-α5 integrin antibodies. N ≥ 3 independent experiments.

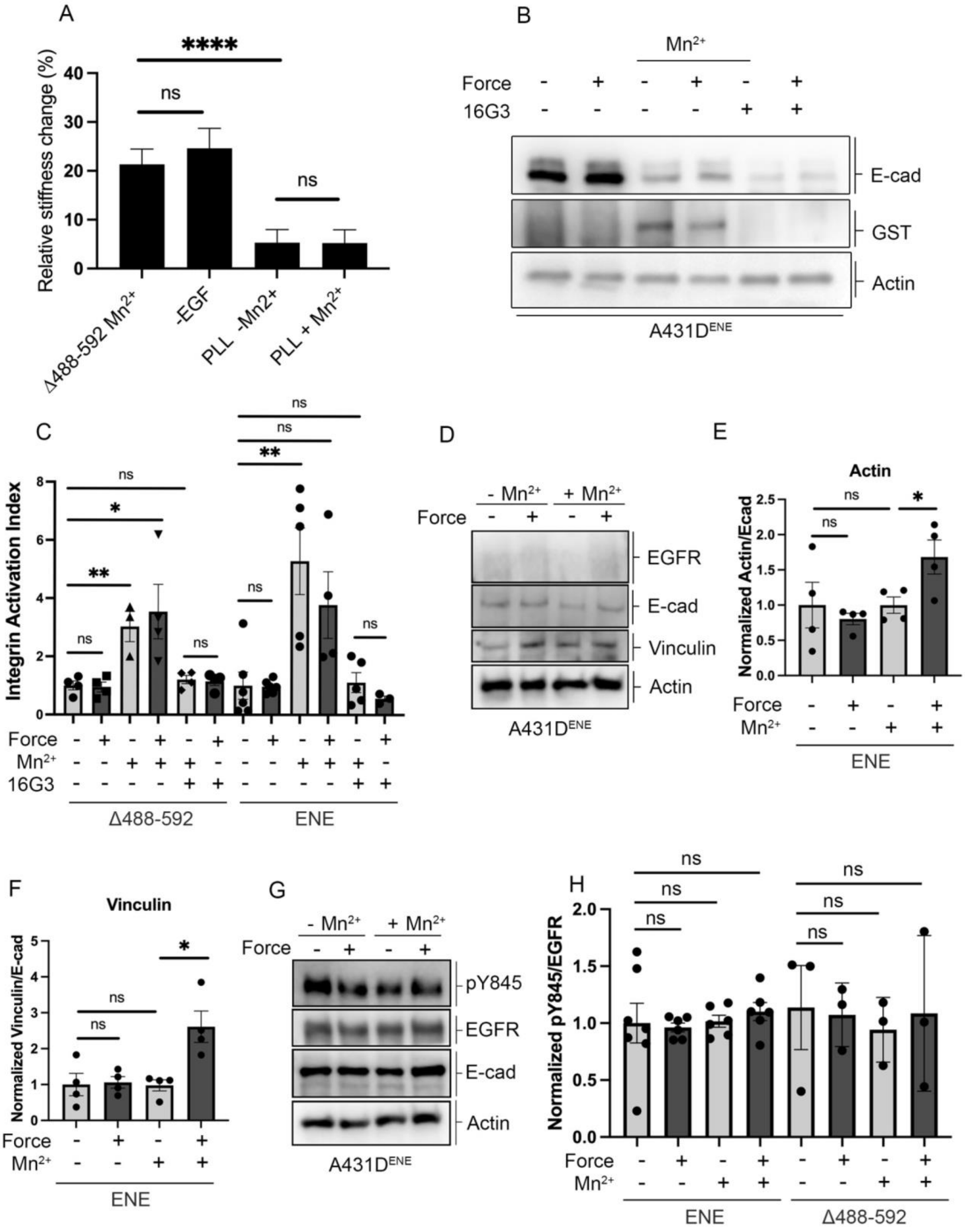

A
Relative stiffness change (%)
****
ns
ns
Δ488-592 Mn2+
-EGF
PLL -Mn2+
PLL + Mn2+
B
Mn2+
Force
16G3
E-cad
GST
Actin
A431D^ENE
C
Integrin Activation Index
Force
Mn2+
16G3
Δ488-592
ENE
D
- Mn2+
+ Mn2+
Force
EGFR
E-cad
Vinculin
Actin
A431D^ENE
E
Actin
Normalized Actin/Ecad
ENE
F
Vinculin
Normalized Vinculin/E-cad
ENE
G
- Mn2+
+ Mn2+
Force
pY845
EGFR
E-cad
Actin
A431D^ENE
H
Normalized pY845/EGFR
ENE
Δ488-592

Fig. S4. (Supplement to Fig. 4). A) Relative percent stiffness change measured with A431-D cells expressing the Δ488-592 mutant. Cells perturbed with E-cad-Fc beads were treated with Mn^{2+} or EGF ($-Mn^{2+}$). Measurements were also done with PLL-bead controls, $\pm Mn^{2+.}$ B) Representative Western blot of GST-Fn_{9-11} uptake (integrin activation index) in ENE expressing A431-D cells. The top heading indicates the application of force (bead twisting), and the second heading indicates 16G3 treatment. Conditions under which integrins were preactivated with Mn^{2+} are indicated by the horizontal bar. C) Bar graph of the quantified integrin activation index. Individual GST intensity is normalized with in -force, -16G3 and - Mn^{2+} condition to reflect integrin activation by each treatment. D) Pull-down measurement with E-cad-beads, ± bead twisting. Measurements were done $\pm Mn^{2+}$. Western blots are shown for EGFR, E-cadherin, vinculin, and actin pulled down with E-cadherin beads under the different conditions. E and F) Bar graphs of the relative Vinculin/E-cad or Actin/E-cad levels pulled down under each condition. Data are normalized by to the unperturbed (-Force, -Mn^{2+}) condition. N = 3 independent experiments. ** $p< 0.005$, * $p<0.05$. G) Western blot results for EGFR phosphorylation, pY845, total EGFR, E-cad, and Actin in A431-D cells expressing WT E-cadherin. Cells were treated with Mn^{2+} and/or Force. H) Bar graph of quantified pY845/EGFR levels for ENE and Δ488-592 mutants. The pY845/EGFR ratio for each condition is normalized to the unperturbed condition (-Force, $-Mn^{2+}$). $N \geq 3$ independent experiments. * $p<0.05$.

Journal of Cell Science • Supplementary information

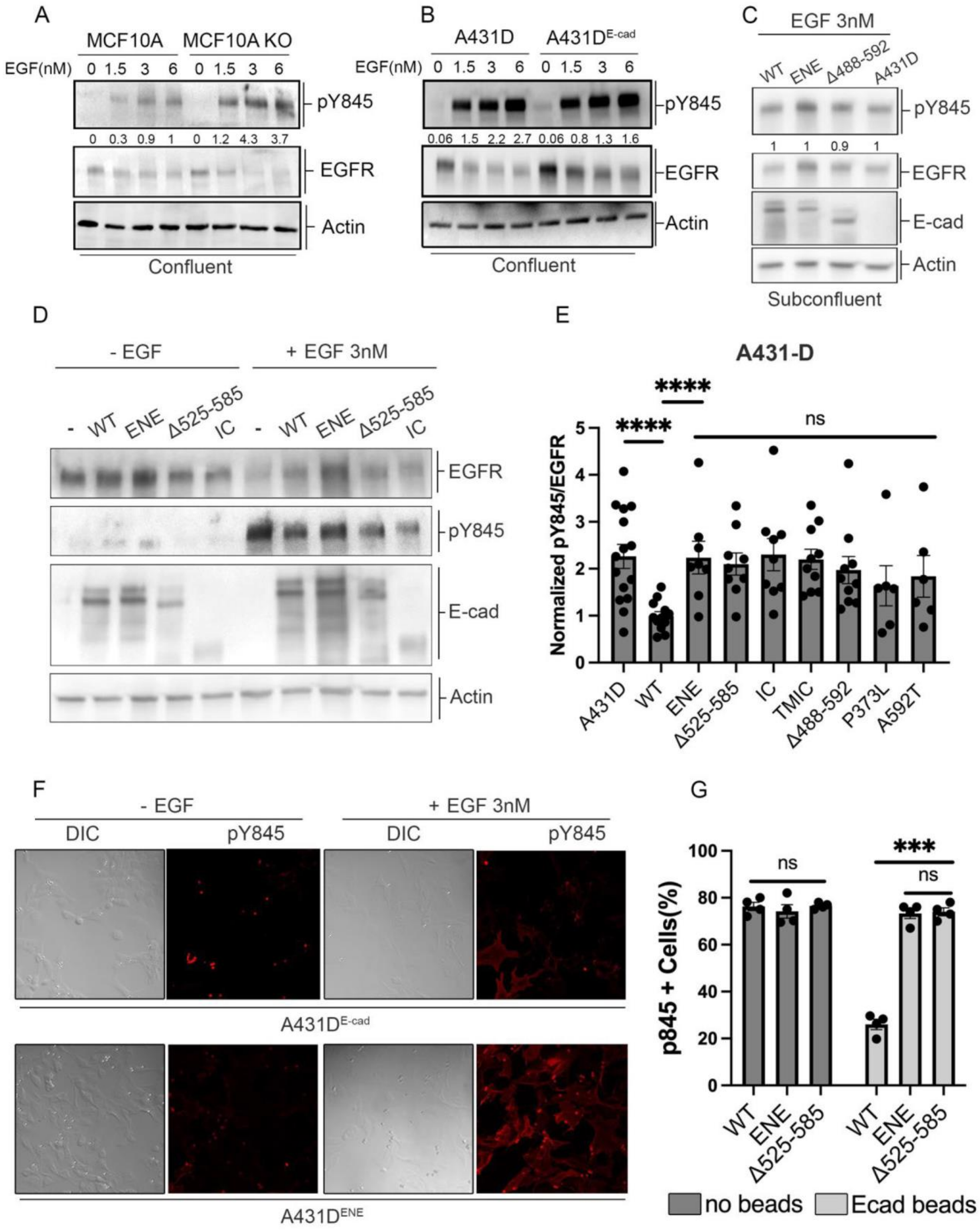

Journal of Cell Science • Supplementary information

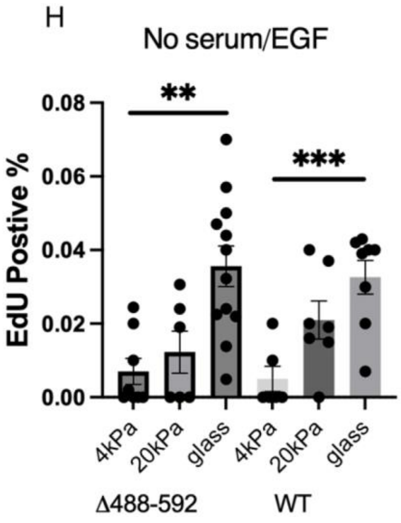

Fig S5. (Supplement to Fig. 5). A) Representative Western blot of pY845, EGFR, and actin in confluent monolayers of MCF10A cells or MCF10A KO cells at 10min after treatment with EGF at the indicated concentrations. B) Representative Western blot of pY845, EGFR, and actin in confluent monolayers of A431-D cells and A431-D cells expressing WT E-cadherin. Measurements were done 10min after treatment with EGF at the indicated concentrations. C) Western blot of pY845, EGFR, E-cad, and actin in sub-confluent monolayers of A431-D or A431-D cells expressing the indicated E-cad mutants. D) Panel shows representative Western blot for pY845 EGFR in cells expressing ENE, Δ525-585, and IC mutants. Cell monolayers were serum starved and treated with 3nM EGF. E) Bar graph quantifying blot intensities. The pY845/EGFR ratio of WT expressing cells is compared with all mutants listed. **** $p<0.0001$, N ≥ 5 independent experiments. F) DIC and pY845 immunofluorescence (red) images of cells with or without E-cadherin-coated beads. G) Percentage of pY845 positive cells with or without bound E-cad beads, after treatment with 3nM EGF. Cells expressed WT E-cadherin or the mutants ENE or Δ525-585. Student's t-test: *** $p<0.001$. N = 4 independent experiments. H) % EdU positive, serum-starved MCF10A KO cells on fibronectin-coated glass and polyacrylamide gels. Cells expressed WT E-cadherin orΔ 488-592 were cultured on fibronectin-coated glass or polyacrylamide gels with Young's moduli of 4 and 20kPa. Data were from N > 6 independent measurements. ** $p< 0.005$, * $p<0.05$.

Journal of Cell Science • Supplementary information

Table S1. A431-D Surface expression of E-cadherin mutants quantified by flow-cytometry.

Cadherin Variants	Protein/µm^2	Number of experiments
WT	31±10	8
ENE	31±7	4
Δ525-585	30±4	4
Δ488-592	30±3	4
A592T	36±3	3
P373L	35±7	2

Table S2. Quantitative change in cell stiffness measured by magnetic twisting cytometry

Cell type	Mutant expressed	Integrin pre-activation	Cell Stiffness Change (mean ± SEM)
A431-D	WT		24 ± 2%
A431-D	PLL control		0 ± 2%
A431-D	IL2R-IC		6 ± 2%
A431-D	IL2R-TMIC		6 ± 2%
A431-D	ENE		6 ± 2%
A431-D	Δ525-585		4 ± 2%
A431-D	Δ488-592		4 ± 2%
A431-D	A592T		14 ± 2%
A431-D	P373L		27% ± 3%
A431-D	ENE	Mn^{2+}	23 ± 4%
A431-D	ENE	Serum starved, Mn^{2+}	28 ± 6%
MCF10A	WT		27% ± 3%
MCF-10A	Δ488-592		10 ± 3%
MCF-10A	A592T		16% ± 3%

Journal of Cell Science • Supplementary information
